# Supplementary material for: The cell cycle regulator GpsB functions as cytosolic adaptor for multiple cell wall enzymes
Source: Nat Commun. 2019 Jan 16;10:261. doi: 10.1038/s41467-018-08056-2 (PMC6335420; doi:10.1038/s41467-018-08056-2)
Supplement: Supplementary file 1 — Supplementary Information [file 41467_2018_8056_MOESM1_ESM.pdf]

Supplementary information for:

The cell cycle regulator GpsB functions as cytosolic adaptor for multiple cell wall enzymes

Robert M. Cleverley[1], Zoe J.Rutter[1], Jeanine Rismondo[2,6], Federico Corona[3,7], Ho-Ching Tiffany Tsui[4], Fuad A. Alatawi[5], Richard A. Daniel[5], Sven Halbedel[2], Orietta Massidda[3,8], Malcolm E. Winkler[4] and Richard J. Lewis[1]

[1]Institute for Cell and Molecular Biosciences, University of Newcastle, Newcastle upon Tyne, United Kingdom, NE2 4HH.

[2]FG11 Division of Enteropathogenic bacteria and Legionella, Robert Koch Institute, Burgstrasse 37, D-38855 Wernigerode, Germany.

[3]Dipartimento di Scienze Chirurgiche, Università di Cagliari, Cagliari, 09100, Italy.

[4]Department of Biology, Indiana University Bloomington, Bloomington, IN 47405, USA.

[5]Centre for Bacterial Cell Biology, Institute for Cell and Molecular Biosciences, University of Newcastle, Newcastle upon Tyne, United Kingdom, NE2 4AX.

[6]Present address: Section of Microbiology and MRC Centre for Molecular Bacteriology and Infection, Imperial College London, London, United Kingdom, SW7 2DD.

[7]Present address: Centre for Bacterial Cell Biology, Institute for Cell and Molecular Biosciences, University of Newcastle, Newcastle upon Tyne, United Kingdom, NE2 4AX.

[8]Present address: Department CIBIO, University of Trento, via Sommarive 9, 38123 Povo, Italy.

Correspondence and requests for materials should be addressed to R.J.L (email: r.lewis@ncl.ac.uk)

Keywords: peptidoglycan / cell division / divisome / penicillin binding protein / GpsB

Supplementary Note 1

By circular dichroism measurements of purified recombinant proteins, wild-type *Bs*PBP1$_{1\text{-}32}$ has α-helical content of 14 %, corresponding to ~5 residues (**Supplementary Figure 1B**), consistent with the 6 α-helical amino acids in the structure of the *Bs*GpsB$_{5\text{-}64}$:*Bs*PBP1$_{1\text{-}17}$ complex (**Figure 1B**, **1C**). *Bs*PBP1$_{1\text{-}32}^{\text{Ala10Pro}}$ is mostly random coil with α-helical content of 2 % (one amino acid) and a >8-fold reduction in binding affinity for *Bs*GpsB$_{1\text{-}68}$. *Bs*PBP1$_{1\text{-}32}^{\text{Ser7Ala}}$ has a slightly higher α-helical content (19%; ~6 amino acids) than the wild-type, presumably because the helix is initiated at Asn6 in this peptide. Asparagine also preferentially occupies the N-cap position in α-helices[1] and promotes α-helix formation when introduced at the N-terminus of model peptides[2]. If *Bs*PBP1$_{1\text{-}32}^{\text{Asn6}}$ was the N-cap, its sidechain would hydrogen bond to the amide nitrogen of *Bs*PBP1$_{1\text{-}32}^{\text{Glu9}}$, negatively impacting on the interaction between its amide and *Bs*GpsB$_{5\text{-}64}^{\text{Asp35}}$, and explaining the >5-fold reduced binding affinity of this mutant.

*Lm*PBPA1$_{1\text{-}15}$ and *Lm*PBPA1$_{1\text{-}15}^{\text{Gln10Pro}}$ are essentially random coil peptides, consistent with the disorder of the *Lm*PBPA1$_{1\text{-}15}$ in the *Bs*GpsB$_{5\text{-}64}^{\text{Lys32Glu}}$:*Lm*PBPA1$_{1\text{-}15}$ structure. The molar ellipticity signal at 222 nm in CD is 40% higher in wild-type *Lm*PBPA1$_{1\text{-}15}$ at TFE concentrations of 40, 60 and 80% than in *Lm*PBPA1$_{1\text{-}15}^{\text{Gln10Pro}}$ (**Supplementary Figure 1D**). Q10 in the *Lm*PBPA1 peptide is completely disordered in the structure of *Bs*GpsB$_{5\text{-}64}^{\text{Lys32Glu}}$:*Lm*PBPA1$_{1\text{-}15}$, (**Figure 2A**), and the impact of *Lm*PBPA1$_{1\text{-}20}^{\text{Gln10Pro}}$ likely reflects an effect on the peptide conformation rather than a loss of contacts to GpsB.

Supplementary Note 2

Proteins with established roles in growth, division and morphogenesis, including the early cell division proteins FtsZ (*lmo2032*), FtsA (*lmo2033*), EzrA (*lmo1594*), ZapA (*lmo1229*) and SepF (*lmo2030*), the late division proteins DivIB (*lmo2034*), DivIC (*lmo0217*), FtsL (*lmo2040*), FtsW (*lmo1071*) as well as the elangosomal proteins MreB (*lmo1548*), MreC (*lmo1547*), MreD (*lmo1546*), MreBH (*lmo1713*), Mbl (*lmo2525*), RodA (*lmo2427*) and RodZ (*lmo1395*) were screened for interaction wth *Lm*GpsB by BACTH. The nucleoid occlusion factor Noc (*lmo2794*), four other high molecular weight penicillin binding proteins (HMW PBPs) PBP A2 (*lmo2229*), PBPB1 (*lmo1438*), PBPB2 (*lmo2039*) and PBPB3 (*lmo0441*) as well as the PG N-deacetylase PgdA (*lmo0415*) were also included. In contrast to *B. subtilis*, which has FtsW, RodA and SpoVE, a sporulation-specific homologue of FtsW/RodA, the *L. monocytogenes* genome contains four additional FtsW/RodA homologues encoded by *lmo0421*, *lmo2428*, *lmo2687* and *lmo2688*. Their function is presently unknown, but they were also included in the screen for GpsB interaction partners.

Supplementary Table 1: Dissociation constants of GpsB:PBP complexes

GpsB binding partner and sequence	mutation	GpsB protein	K_d µM
TAMRA-*Bs*PBP1$_{1-32}$ *GS*M$_1$*A*DQFNSREARRKAN*C*KSSPSPKKGKKRKKGG$_{32}$	Wildtype	*Bs*GpsB$_{1-68}$	120 ± 10
	Ser7Ala		>700
	Arg8Ala		>1700
	Arg8Lys		>2000
	Ala10Pro		>1000
	Arg11Ala		>600
	Arg11Lys		390 ± 20
	Arg28Ala		90 ± 10
	Wildtype	*Bs*GpsB$_{1-68}^{Glu17Ala}$	>1300
	Wildtype	*Bs*GpsB$_{1-68}^{Tyr25Phe}$	>2000
	Wildtype	*Bs*GpsB$_{1-68}^{Asp31Ala}$	>1900
	Wildtype	*Bs*GpsB$_{1-68}^{Asp35Ala}$	>2000
	Wildtype	*Bs*GpsB	160 ± 10
Fluorescein-*Bs*PBP1$_{1-32}$ *GS*M$_1$*A*DQFNSREARRKANSKSSPSPKKGKKRKK*C*G$_{32}$	Wildtype	*Bs*GpsB$_{1-68}$	100 ± 10
	Arg8Ala		>500
	Ala10Pro		>500
	Arg28Ala		130 ± 20
Fluorescein-*Lm*PBPA1$_{1-20}$ *GS*M$_1$ADKPQTRSQYRNKQSGG*C*K$_{20}$	Wildtype	*Bs*GpsB$_{1-68}^{Lys32Glu}$	190 ± 40
	Arg8Ala		>3000
	Arg8AlaSer16Arg		>2000
	Gln10Pro		>1500
	Tyr11Ala		430 ± 40
	Arg12Ala		800 ± 40
	Wildtype	*Lm*GpsB$_{1-73}$	200 ± 20
TAMRA-*Sp*PBP2a$_{23-45}$ *GSM*D$_{23}$SDSTILRRSRSDRKKLAQV*C*PI$_{45}$	Wildtype	*Sp*GpsB$_{1-63}$	80 ± 20
	Arg31Lys		150 ± 10
	Arg33Lys		360 ± 30
	Arg31LysArg33Lys		>2000
	Ser32Ala		210 ± 15
	Arg33Ala		530 ± 50
	Arg36Ala		270 ± 20
	Wildtype	*Sp*GpsB$_{1-63}^{Asp33Ala}$	>3000
Fluorescein-*Sp*PBP2b$_{1-17}$ Fluorescein-GM$_1$RLICMRKFNSHSIPIR$_{17}$	Wildtype	*Sp*GpsB$_{1-63}$	>3600
Fluorescein-*Sp*PBP2x$_{1-29}$ *GSGSG*M$_1$*E*WTKRVIRYATKNRKSPAENRRRVGKSL*CS*	Wildtype	*Sp*GpsB$_{1-63}$	370 ± 30
Fluoroescein-*Bs*YpbE$_{1-21}$ Fluoroescein-J$_1$TNJSRVERRKAQNLYEDQNA$_{21}$	Wildtype	*Bs*GpsB$_{1-68}$	13 ± 1
	Wildtype	*Bs*GpsB$_{1-68}^{Tyr25Phe}$	>500
	Wildtype	*Bs*GpsB$_{1-68}^{Asp31Ala}$	>500
Fluoroescein-*Bs*YrrS$_{1-18}$ *GS*M$_1$GNNQSRYENRDKRRKAN$_{18}$*CG*	Wildtype	*Bs*GpsB$_{1-68}$	430 ± 20
	Wildtype	*Bs*GpsB$_{1-68}^{Tyr25Phe}$	>3000
	Wildtype	*Bs*GpsB$_{1-68}^{Asp31Ala}$	>3000

Dissociation constants for the interaction of PBP cytoplasmic mini-domain peptides and other binding partners with N-terminal domains of GpsB proteins measured by fluorescence polarization.

The subscripts in the peptide sequences represent the residue numbers in the relevant protein; the italics denote non-native residues at the termini resulting from the recombinant method used to produce peptides. All peptides were labelled with a TAMRA or fluorescein fluorophore at the underlined cysteine at the C-terminus in all cases except for *Bs*YpbE$_{1\text{-}21}$, *Sp*PBP2b$_{1\text{-}17}$ and *Bs*PBP1$_{1\text{-}32}$. The *Bs*YpbE$_{1\text{-}21}$ and *Sp*PBP2b$_{1\text{-}17}$ peptides were labelled at the N-terminus with fluorescein; in the former peptide norleucine (abbreviated with a ‘J’) replaced the naturally-occurring methionine to avoid sulphoxidation during synthesis caused by proximity of the fluorophore. In *Bs*PBP1$_{1\text{-}32}$, cysteine replaced PBP1$^{\text{Ser16}}$, which is remote from the protein:peptide interface in the structure of the *Bs*GpsB$_{5\text{-}64}$:*Bs*PBP1$_{1\text{-}17}$ complex. A *Bs*PBP1$_{1\text{-}32}$ peptide labelled at its C-terminus (at residue 31) had the same affinity for *Bs*GpsB$_{1\text{-}68}$ in FP experiments[3] as the equivalent residue-16 labelled peptide, above. The affinity of 31-labelled peptides also has the same pattern of sensitivity to R8A, A10P and R28A point mutations as 16-labelled peptides.

The affinities measured herein by FP with soluble protein fragments very likely translate to higher affinities in bacterial cells since the FP measurements do not take into account likely avidity effects that would enhance the affinity of the interaction if full-length GpsB proteins were used in combination with their full-length integral membrane protein interaction partners in the context of a biological membrane. Affinity measurements in solution of components that ordinarily interact only in the context of a membrane have indeed been found to be misleading[4].

Supplementary Table 2. Δ*pbp1a*::P_c-*erm* transformation efficiencies and colony sizes

Recipient strain	*pbp2a* genotype of recipient strain	Number of Δ*pbp1a*::P_c-*erm* transformants at 24 h (colony size after streaking; strain)[a]
IU1824	*pbp2a*$^{+}$	>500 (medium[b], IU13444)
IU13256	Δ*pbp2a*	0
IU13258	Δ2-49[c]	0
IU14256	R31A	> 500 (medium, IU14294)
IU14259	R31K R33K	> 500 (medium, IU14296)
IU14263	R33A	> 500 (medium, IU14298)
IU14400	R31A S32A R36A	>500 (medium, IU14416)
IU13180	Δ32-37 (ΔSRSDRK)	>500 (medium, IU13446)
IU14396	Δ31-36	>500 (medium, IU14414)
IU14394	Δ29-36	>500 (medium, IU14412)
IU13298	Δ27-38	>500 (medium, IU13448)
IU13301	Δ26-45	>500 (small, IU13450)
IU14502	Δ2-22	>500 (small, IU14516)

[a]The recipient *S. pneumoniae* strains are described in **Supplementary Table 3**. The transformation and visualization of colonies was performed as described in the **Supplementary Materials and Methods**. The numbers of colonies are normalized to 1 mL of transformation mixture.

[b]The streaked colonies of strains with the Δ*pbp1a*::P_c-*erm* genotype were smaller than isogenic *pbp1a*$^{+}$ parent (IU1824).

[c]The cytoplasmic region of *Sp*PBP2a comprises the first 56 residues.

Supplementary Table 3: Strains used in this study

**name**	**relevant characteristics / genotype**	**Ab^{R*}**	**source/ reference**
***L. monocytogenes* strains**			
EGD-e	wildtype, serovar 1/2a strain	None	lab collection
LMJR19	Δ*gpsB* (*lmo1888*)	None	3
LMS57	Δ*pbpA1* (*lmo1892*)	None	5
LMS64	Δ*pbpA2* (*lmo2229*)	None	5
LMS211	*pbpA1*$^{\Delta N}$	None	This work
LMS215	*pbpA1 T7A*	None	This work
LMS216	*pbpA1 R8A*	None	This work
LMS217	*pbpA1 Y11A*	None	This work
LMS218	*pbpA1 R12A*	None	This work
LMS219	*pbpA1 T7A* Δ*pbpA2*	None	This work
LMS220	*pbpA1 R8A* Δ*pbpA2*	None	This work
LMS221	*pbpA1 Y11A* Δ*pbpA2*	None	This work
LMS222	*pbpA1 R12A* Δ*pbpA2*	None	This work
LMS229	*pbpA1 R8A R12A*	None	This work
LMS230	*pbpA1Q10P*	None	This work
LMS232	*pbpA1 R8A R12A* Δ*pbpA2*	None	This work
LMS233	*pbpA1 Q10P* Δ*pbpA2*	None	This work
***S. pneumoniae* strains**			
IU1824[c]	D39 *rpsL1* Δ*cps2A'-cps2H'* = D39 *rpsL1* □*cps*	St	6
IU1945	D39 Δ*cps2A'-cps2H'*= D39 Δ*cps*	None	6
E177	D39 Δ*cps* Δ*pbp1a*::P$_c$-*erm*	E	7
K166	D39 Δ*cps* Δ*pbp2a*::P$_c$-[*kan-rpsL*$^+$]	K	7
IU4888	D39 Δ*cps* Δ*gpsB*<>*aad9*//Δ*bgaA*::*kan*-t1t2-P$_{fcsK}$-*gpsB*$^+$	K Sp	8
IU4970	D39 Δ*cps mreC*-L-FLAG^3-P$_c$-*erm*	E	9
IU5458	D39 Δ*cps gpsB*-L-FLAG^3-P$_c$-*erm*	E	8
IU5838	D39 Δ*cps gpsB*-FLAG-P$_c$-*erm*	E	8
IU6442	D39 Δ*cps* Δ*gpsB*<>*aad9 phpP* (G229D)	Sp	10
IU6810	D39 Δ*cps ezrA*-HA-Pc-*kan*	K	10
IU6819	D39 Δ*cps pbp2x*-FLAG^3-P$_c$-*erm*	E	11
IU7434	D39 Δ*cps stkP*-FLAG^2-P$_c$-*erm*	E	11
IU7853	D39 Δ*cps rpsL1* Δ*pbp2a*::P$_c$-[*kan-rpsL*$^+$] (IU1824 X Δ*pbp2a*::P$_c$-[*kan-rpsL*$^+$] from K166)	K	This work
IU8122	D39 Δ*cps* Δ*bgaA*::*tet*-P$_{Zn}$-RBS^{ftsA}-*ftsZ*$^+$	T	12
IU8496	D39 Δ*cps* Δ*divIVA*::P$_c$-*erm* (IU1945 X fusion Δ*divIVA*:: P$_c$-*erm*)	E	This work
IU11051	D39 Δ*cps gpsB*$^+$-P$_c$-*erm* (IU1945 X fusion *gpsB*$^+$-P$_c$-*erm*)	E	This work
IU11286	D39 Δ*cps* Δ*bgaA*::*tet*-P$_{Zn}$-RBS^{ftsA}-*gpsB*$^+$ (IU1945 X fusion Δ*bgaA*::*tet*-P$_{Zn}$- *gpsB*$^+$)	T	This work
IU11314	D39 Δ*cps gpsB*-L-FLAG^3-P$_c$-*erm pbp2x*-HA-P$_c$-*kan*	E K	10

**name**	**relevant characteristics / genotype**	**Ab^{R*}**	**source/ reference**
IU11316	D39 Δ*cps gpsB*-L-FLAG^3-P_c-*erm pbp2b*-HA-P_c-*kan*	E K	10
IU11388	D39 Δ*cps* Δ*gpsB*<>*aad9*//Δ*bgaA*::*tet*-P_{Zn}-RBS^{ftsA}-*gpsB*$^+$ (IU11286 X Δ*gpsB*<>*aad9* from IU4888)	T Sp	This work
IU11488	D39 Δ*cps gpsB*$^+$-P_c-*erm*//Δ*bgaA*::*tet*-P_{Zn}-RBS^{ftsA}-*gpsB*$^+$ (IU11286 X *gpsB*$^+$-P_c-*erm* from IU11051)	T E	This work
IU11880	D39 Δ*cps ezrA*-HA-P_c-*kan*-*pbp2x*-FLAG^3-P_c-*erm* (IU6810 X *pbp2x*-FLAG^3-P_c-*erm* from IU6819)	E K	This work
IU12077	D39 Δ*cps ezrA*-HA-P_c-*kan*-stkP-FLAG^2-P_c-*erm* (IU7434 X *ezrA*-HA-P_c-*kan* from IU6810)	E K	This work
IU12361	D39 Δ*cps gpsB* D29A-P_c-*erm*//Δ*bgaA*::*tet*-P_{Zn}-RBS^{ftsA}-*gpsB*$^+$ (IU11388 X fusion *gpsB* D29A-P_c-*erm*)	T E	This work
IU12363	D39 Δ*cps gpsB* D33A-P_c-*erm*//Δ*bgaA*::*tet*-P_{Zn}-RBS^{ftsA}-*gpsB*$^+$ (IU11388 X fusion *gpsB* D33A-P_c-*erm*)	T E	This work
IU12440	D39 Δ*cps gpsB* Y23A-P_c-*erm*//Δ*bgaA*::*tet*-P_{Zn}-RBS^{ftsA}-*gpsB*$^+$ (IU11388 X fusion *gpsB* Y23A-P_c-*erm*)	T E	This work
IU12612	D39 Δ*cps gpsB* V28A-P_c-*erm*//Δ*bgaA*::*tet*-P_{Zn}-RBS^{ftsA}-*gpsB*$^+$ (IU11388 X fusion *gpsB* V28A-P_c-*erm*)	T E	This work
IU12615	D39 Δ*cps gpsB* L32A-P_c-*erm*//Δ*bgaA*::*tet*-P_{Zn}-RBS^{ftsA}-*gpsB*$^+$ (IU11388 X fusion *gpsB* L32A-P_c-*erm*)	T E	This work
IU12788	D39 Δ*cps rpsL1* Δ*bgaA*::*kan*-P_{Zn}-RBS^{ftsA}-*khpA*$^+$	K	12
IU13121	D39 Δ*cps gpsB* I36A-P_c-*erm*//Δ*bgaA*::*tet*-P_{Zn}-RBS^{ftsA}-*gpsB*$^+$ (IU11388 X fusion *gpsB* I36A-P_c-*erm*)	T E	This work
IU13141	D39 Δ*cps gpsB* D29A-P_c-*erm*//Δ*bgaA*::*tet*-P_{Zn}-RBS^{ftsA}-*gpsB*$^+$ (IU11388 X *gpsB* D29A-P_c-*erm* from IU12361)	T E	This work
IU13180	D39 Δ*cps rpsL1 pbp2a* Δ32-37 (IU7853 X fusion *pbp2a* Δ32-37)	St	This work
IU13256	D39 Δ*cps rpsL1* Δ*pbp2a* (IU7853 X fusion Δ*pbp2a* markerless)	St	This work
IU13258	D39 Δ*cps rpsL1 pbp2a* Δ2-49 (IU7853 X fusion *pbp2a* Δ2-49)	St	This work
IU13298	D39 Δ*cps rpsL1 pbp2a* Δ27-38 (IU7853 X fusion *pbp2a* Δ27-38)	St	This work
IU13301	D39 Δ*cps rpsL1 pbp2a* Δ26-45 (IU7853 X fusion *pbp2a* Δ26-45)	St	This work
IU13364	D39 Δ*cps gpsB* Y23A-FLAG-P_c-*erm*//Δ*bgaA*::*tet*-P_{Zn}-RBS^{ftsA}-*gpsB*$^+$ (IU11388 X fusion *gpsB* Y23A-FLAG-P_c-*erm*)	T E	This work
IU13366	D39 Δ*cps gpsB* V28A-FLAG-P_c-*erm*//Δ*bgaA*::*tet*-P_{Zn}-RBS^{ftsA}-*gpsB*$^+$ (IU11388 X fusion *gpsB* V28A-FLAG-P_c-*erm*)	T E	This work
IU13368	D39 Δ*cps gpsB* D29A-FLAG-P_c-*erm*//Δ*bgaA*::*tet*-P_{Zn}-RBS^{ftsA}-*gpsB*$^+$ (IU11388 X fusion *gpsB* D29A-FLAG-P_c-*erm*)	T E	This work
IU13370	D39 Δ*cps gpsB* L32A-FLAG-P_c-*erm*//Δ*bgaA*::*tet*-P_{Zn}-RBS^{ftsA}-*gpsB*$^+$ (IU11388 X fusion *gpsB* L32A-FLAG-P_c-*erm*)	T E	This work
IU13372	D39 Δ*cps gpsB* D33A-FLAG-P_c-*erm*//Δ*bgaA*::*tet*-P_{Zn}-RBS^{ftsA}-*gpsB*$^+$ (IU11388 X fusion *gpsB* D33A-FLAG-P_c-*erm*)	T E	This work
IU13374	D39 Δ*cps gpsB* I36A-FLAG-P_c-*erm*//Δ*bgaA*::*tet*-P_{Zn}-RBS^{ftsA}-*gpsB*$^+$ (IU11388 X fusion *gpsB* I36A-FLAG-P_c-*erm*)	T E	This work
IU13442	D39 Δ*cps gpsB*-FLAG-P_c-*erm*//Δ*bgaA*::*tet*-P_{Zn}-RBS^{ftsA}-*gpsB*$^+$ (IU11388 X *gpsB*-FLAG-P_c-*erm* from IU5838)	T E	This work
IU13444	D39 Δ*cps rpsL1* Δ*pbp1a*::P_c-*erm* (IU1824 X Δ*pbp1a*::P_c-*erm* from E177)	St E	This work
IU13446	D39 Δ*cps rpsL1 pbp2a* Δ32-37 Δ*pbp1a*::P_c-*erm* (IU13180 X Δ*pbp1a*::P_c-*erm* from E177)	St E	This work
IU13448	D39 Δ*cps rpsL1 pbp2a* Δ27-38 Δ*pbp1a*::P_c-*erm* (IU13298 X Δ*pbp1a*::P_c-*erm* from E177)	St E	This work
IU13450	D39 Δ*cps rpsL1 pbp2a* Δ26-45 Δ*pbp1a*::P_c-*erm* (IU13301 X Δ*pbp1a*::P_c-*erm* from E177)	St E	This work
IU14256	D39 Δ*cps rpsL1 pbp2a* R31A (IU7853 X fusion *pbp2a* R31A)	St	This work
IU14259	D39 Δ*cps rpsL1 pbp2a* R31K R33K (IU7853 X fusion *pbp2a* R31K R33K)	St	This work
IU14263	D39 Δ*cps rpsL1 pbp2a* R33A (IU7853 X fusion *pbp2a* R33A)	St	This work
IU14294	D39 Δ*cps rpsL1 pbp2a* R31A Δ*pbp1a*::P_c-*erm* (IU14256 X Δ*pbp1a*::P_c-*erm* from E177)	St E	This work
IU14296	D39 Δ*cps rpsL1 pbp2a* R31K R33K Δ*pbp1a*::P_c-*erm* (IU14259 X Δ*pbp1a*::P_c-*erm* from E177)	St E	This work
IU14298	D39 Δ*cps rpsL1 pbp2a* R33A Δ*pbp1a*::P_c-*erm* (IU14263 X Δ*pbp1a*::P_c-*erm* from E177)	St E	This work
IU14318	D39 Δ*cps rpsL1* Δ*bgaA*::*kan*-P_{Zn}-RBS^{ftsA}-*pbp2a*$^+$ (IU1824 X fusion Δ*bgaA*:: *kan*-P_{Zn}-RBS^{ftsA}-*pbp2a*$^+$)	St K	This work
IU14365	D39 Δ*cps rpsL1* Δ*pbp2a* markerless//Δ*bgaA*::*kan*-P_{Zn}-RBS^{ftsA}-*pbp2a*$^+$ (IU13256 X Δ*bgaA*::*kan*-P_{Zn}-RBS^{ftsA}-*pbp2a*$^+$ from IU14318)	St K	This work
IU14381	D39 Δ*cps rpsL1* Δ*pbp2a* markerless//Δ*bgaA*::*kan*-P_{Zn}-RBS^{ftsA}-*pbp2a*$^+$ Δ*pbp1a*::P_c-*erm* (IU14365 X Δ*pbp1a*::P_c-*erm* from E177)	St K E	This work
IU14383	D39 Δ*cps* Δ*gpsB*<>*aad9*//Δ*bgaA*::*tet*-P_{Zn}-RBS^{ftsA}-*gpsB*$^+$ Δ*pbp1a*::P_c-*erm* (IU11388 X Δ*pbp1a*::P_c-*erm* from E177)	T Sp E	This work

name	relevant characteristics / genotype	Ab^{R*}	source/ reference
IU14394	D39 Δ*cps rpsL1 pbp2a* Δ29-36 (IU7853 X fusion *pbp2a* Δ29-36)	St	This work
IU14396	D39 Δ*cps rpsL1 pbp2a* Δ31-36 (IU7853 X fusion *pbp2a* Δ31-36)	St	This work
IU14400	D39 Δ*cps rpsL1 pbp2a* R31A S32A R36A(IU7853 X fusion *pbp2a* R31A S32A R36A)	St	This work
IU14412	D39 Δ*cps rpsL1 pbp2a* Δ29-36 Δ*pbp1a*::P_c-*erm* (IU14394 X Δ*pbp1a*::P_c-*erm* from E177)	St E	This work
IU14414	D39 Δ*cps rpsL1 pbp2a* Δ31-36 Δ*pbp1a*::P_c-*erm* (IU14396 X Δ*pbp1a*::P_c-*erm* from E177)	St E	This work
IU14416	D39 Δ*cps rpsL1 pbp2a* R31A S32A R36A Δ*pbp1a*::P_c-*erm* (IU14400 X Δ*pbp1a*::P_c-*erm* from E177)	St E	This work
IU14502	D39 Δ*cps rpsL1 pbp2a* Δ2-22 (IU7853 X fusion *pbp2a* Δ2-22)	St	This work
IU14516	D39 Δ*cps rpsL1 pbp2a* Δ2-22 Δ*pbp1a*::P_c-*erm* (IU14502 X Δ*pbp1a*::P_c-*erm* from E177)	St E	This work

*Ab^R relates to the antibiotic resistance marker used, St = streptomycin, E = erythromycin, K = kanamycin, T = tetracyclin, Sp = spectinomycin

Supplementary Table 4: Plasmids used in this study

name	relevant characteristics	Two-hybrid construct	source/ reference
**General**			
pMAD	*bla erm bgaB*		13
pKNT25	*kan* P_{lac}-*cya(T25)*	T25	14
pUT18	*amp* P_{lac}-*cya(T25)*	T18	14
pKT25	*kan* P_{lac}-*cya(T25)*	T25	14
pUT18C	*amp* P_{lac}-*cya(T18)*	T18	14
pKT25_*zip*	*kan* P_{lac}-*cya(T25)_zip*	T25-Zip	14
pUT18C_*zip*	*amp* P_{lac}-*cya(T18)_zip*	T18-Zip	14
***B. subtilis* BACTH**			
pFA101	*kan* P_{lac}-*cya(T25)-ypbE*	T25-*Bs*YpbE	This work
pFA103	*kan* P_{lac}-*cya(T25)-rodZ*	T25-*Bs*RodZ	This work
pFA104	*kan* P_{lac}-*cya(T25)-yrrS*	T25-*Bs*YrrS	This work
pFA105	*bla* P_{lac}-*cya(T18)-ypbE*	T18-*Bs*YpbE	This work
pFA107	*bla* P_{lac}-*cya(T18)-rodZ*	T18-*Bs*RodZ	This work
pFA108	*bla* P_{lac}-*cya(T18)-yrrS*	T18-*Bs*YrrS	This work
pKT25-yrrR	*kan* P_{lac}-*cya(T25)-yrrR*	T25-*Bs*YrrR	This work
pUT18C-yrrR	*bla* P_{lac}-*cya(T18)-yrrR*	T18-*Bs*YrrR	This work
pKT25-gpsB	*kan* P_{lac}-*cya(T25)-gpsB*	T25-*Bs*GpsB	15
pUT18C-*gpsB*	*bla* P_{lac}-*cya(T18)-gpsB*	T18-*Bs*GpsB	15
pKT25-*gpsB'*	*kan* P_{lac}-*cya(T25)-gpsB'*$_{1\text{-}65}$	T25-*Bs*GpsB$_{1\text{-}65}$	15
pUT18C-*gpsB'*	*bla* P_{lac}-*cya(T18)-gpsB'*$_{1\text{-}65}$	T18-*Bs*GpsB$_{1\text{-}65}$	15
pKT25-'*gpsB*	*kan* P_{lac}-*cya(T25)-'gpsB*$_{66\text{-}98}$	T25-*Bs*GpsB$_{66\text{-}98}$	15
pUT18C-'*gpsB*	*bla* P_{lac}-*cya(T18)-'gpsB*$_{66\text{-}98}$	T18-*Bs*GpsB$_{66\text{-}98}$	15
pKT25-ponA	*kan* P_{lac}-*cya(T25)-ponA*	T25-*Bs*PBP1	15
pUT18C-ponA	*bla* P_{lac}-*cya(T18)-ponA*	T18-*Bs*PBP1	15
***L. monocytogenes* BACTH**			
pJR233	*kan* P_{lac}-*cya(T25)-mreBH(lmo1713)*	T25-*Lm*MreBH	This work
pJR236	*kan* P_{lac}-*mreBH-cya(T25)*	*Lm*MreBH-T25	This work
pJR242	*kan* P_{lac}-*cya(T25)-mreC(lmo1547)*	T25-*Lm*MreC	This work
pJR243	*kan* P_{lac}-*mreC-cya(T25)*	*Lm*MreC-T25	This work
pJR250	*kan* P_{lac}-*cya(T25)-pbpA2(lmo2229)*	T25-*Lm*PBPA2	This work
pSH236	*kan* P_{lac}-*cya(T25)-pbpB1(lmo1438)*	T25-*Lm*PBPB1	This work
pSH235	*kan* P_{lac}-*cya(T25)-pbpB2(lmo2039)*	T25-*Lm*PBPB2	This work
pSH237	*kan* P_{lac}-*cya(T25)-pbpB3(lmo0441)*	T25-*Lm*PBPB3	This work
pSH437	*kan* P_{lac}-*cya(T25)-pbpA1*$_{\Delta GT\text{-}\Delta TP}$	T25-*Lm*PBPA1$_{\Delta GT\text{-}\Delta TP}$	16
pSH484	*bla* P_{lac}-*gpsB*$^{\Delta N(202\text{-}339)}$-*cya(T18)*	*Lm*GpsB$_{\Delta N}$-T18	This work
pSH485	*kan* P_{lac}-*cya(T25)-pbpA1*$_{\Delta GT\text{-}\Delta TP}^{T7A}$	T25-*Lm*PBPA1$_{\Delta GT\text{-}\Delta TP}^{T7A}$	This work
pSH486	*kan* P_{lac}-*cya(T25)-pbpA1*$_{\Delta GT\text{-}\Delta TP}^{R8A}$	T25-*Lm*PBPA1$_{\Delta GT\text{-}\Delta TP}^{R8A}$	This work
pSH487	*kan* P_{lac}-*cya(T25)-pbpA1*$_{\Delta GT\text{-}\Delta TP}^{Y11A}$	T25-*Lm*PBPA1$_{\Delta GT\text{-}\Delta TP}^{Y11A}$	This work
pSH488	*kan* P_{lac}-*cya(T25)-pbpA1*$_{\Delta GT\text{-}\Delta TP}^{R12A}$	T25-*Lm*PBPA1$_{\Delta GT\text{-}\Delta TP}^{R12A}$	This work
pSH489	*kan* P_{lac}-*cya(T25)-pbpA1*$_{\Delta GT\text{-}\Delta TP}^{K14A}$	T25-*Lm*PBPA1$_{\Delta GT\text{-}\Delta TP}^{K14A}$	This work
pSH490	*kan* P_{lac}-*cya(T25)-pbpA1*$_{\Delta GT\text{-}\Delta TP}^{K20A}$	T25-*Lm*PBPA1$_{\Delta GT\text{-}\Delta TP}^{K20A}$	This work
pSH491	*kan* P_{lac}-*cya(T25)-pbpA1*$_{\Delta GT\text{-}\Delta TP}^{K21A}$	T25-*Lm*PBPA1$_{\Delta GT\text{-}\Delta TP}^{K21A}$	This work
pSH492	*kan* P_{lac}-*cya(T25)-pbpA1*$_{\Delta GT\text{-}\Delta TP}^{K22A}$	T25-*Lm*PBPA1$_{\Delta GT\text{-}\Delta TP}^{K22A}$	This work
pSH493	*kan* P_{lac}-*cya(T25)-pbpA1*$_{\Delta GT\text{-}\Delta TP}^{K25A}$	T25-*Lm*PBPA1$_{\Delta GT\text{-}\Delta TP}^{K25A}$	This work
pSH494	*kan* P_{lac}-*cya(T25)-pbpA1*$_{\Delta GT\text{-}\Delta TP}^{R26A}$	T25-*Lm*PBPA1$_{\Delta GT\text{-}\Delta TP}^{R26A}$	This work
pSH495	*kan* P_{lac}-*cya(T25)-pbpA1*$_{\Delta GT\text{-}\Delta TP}^{K28A}$	T25-*Lm*PBPA1$_{\Delta GT\text{-}\Delta TP}^{K28A}$	This work
pSH496	*kan* P_{lac}-*cya(T25)-pbpA1*$_{\Delta GT\text{-}\Delta TP}^{R29A}$	T25-*Lm*PBPA1$_{\Delta GT\text{-}\Delta TP}^{R29A}$	This work
***S. pneumoniae* BACTH**			
pKNT25_*gpsB*	*kan* P_{lac}-*gpsB-cya(T25)*	*Sp*GpsB-T25	10
pUT18_*gpsB*	*amp* P_{lac}-*gpsB-cya(T18)*	*Sp*GpsB-T18	10
pFC101	*kan* P_{lac}-*gpsB*^{Y23A}-*cya(T25)*	*Sp*GpsB^{Y23A}-T25	This work
pFC102	*amp* P_{lac}-*gpsB*^{Y23A}-*cya(T18)*	*Sp*GpsB^{Y23A}-T18	This work
pFC103	*kan* P_{lac}-*gpsB*^{V28A}-*cya(T25)*	*Sp*GpsB^{V28A}-T25	This work
pFC104	*amp* P_{lac}-*gpsB*^{V28A}-*cya(T18)*	*Sp*GpsB^{V28A}-T18	This work
pFC105	*kan* P_{lac}-*gpsB*^{D29A}-*cya(T25)*	*Sp*GpsB^{D29A}-T25	This work
pFC106	*amp* P_{lac}-*gpsB*^{D29A}-*cya(T18)*	*Sp*GpsB^{D29A}-T18	This work

name	relevant characteristics	Two-hybrid construct	source/ reference
pFC107	*kan* P_{lac}-*gpsB*^{L32A}-*cya(T25)*	*Sp*GpsB^{L32A}-T25	This work
pFC108	*amp* P_{lac}-*gpsB*^{L32A}-*cya(T18)*	*Sp*GpsB^{L32A}-T18	This work
pFC109	*kan* P_{lac}-*gpsB*^{D33A}-*cya(T25)*	*Sp*GpsB^{D33A}-T25	This work
pFC110	*amp* P_{lac}-*gpsB*^{D33A}-*cya(T18)*	*Sp*GpsB^{D33A}-T18	This work
pFC111	*kan* P_{lac}-*gpsB*^{I36A}-*cya(T25)*	*Sp*GpsB^{I36A}-T25	This work
pFC112	*amp* P_{lac}-*gpsB*^{I36A}-*cya(T18)*	*Sp*GpsB^{I36A}-T18	This work
pFC113	*kan* P_{lac}-*cya(T25)-mreC*	T25-*Sp*MreC	This work
pFC114	*amp* P_{lac}-*cya(T18)-mreC*	T18-*Sp*MreC	This work
pFC115	*kan* P_{lac}-*cya(T25)-pbp2a*	T25-*Sp*PBP2a	This work
pFC116	*amp* P_{lac}-*cya(T18)-pbp2a*	T18-*Sp*PBP2a	This work
pFC117	*kan* P_{lac}-*cya(T25)-pbp2a*$_{\Delta 32\text{-}37}$	T25-*Sp*PBP2a$_{\Delta 32\text{-}37}$	This work
pFC119	*kan* P_{lac}-*cya(T25)-pbp2a*$_{\Delta 27\text{-}38}$	T25-*Sp*PBP2a$_{\Delta 27\text{-}38}$	This work
pFC121	*kan* P_{lac}-*cya(T25)-pbp2a*$_{\Delta 26\text{-}45}$	T25-*Sp*PBP2a$_{\Delta 26\text{-}45}$	This work
pFC123	*kan* P_{lac}-*cya(T25)-pbp1a*	T25-*Sp*PBP1a	This work
pFC124	*amp* P_{lac}-*cya(T18)-pbp1a*	T18-*Sp*PBP1a	This work
pFC125	*kan* P_{lac}-*cya(T25)-pbp2b*	T25-*Sp*PBP2b	This work
pFC126	*amp* P_{lac}-*cya(T18)-pbp2b*	T18-*Sp*PBP2b	This work
pFC127	*kan* P_{lac}-*cya(T25)-pbp2x*	T25-*Sp*PBP2x	This work
pFC128	*amp* P_{lac}-*cya(T18)-pbp2x*	T18-*Sp*PBP2x	This work
L. monocytogenes **fosfomycin sensitivity**			
pSH497	*bla erm bgaB-recU-pbpA1´(lmo1892)*		This work
pSH503	*bla erm bgaB-´recU-pbpA1´*$_{\Delta N}$		This work
pSH504	*bla erm bgaB-´recU-pbpA1´*^{T7A}		This work
pSH505	*bla erm bgaB-´recU-pbpA1´*^{R8A}		This work
pSH506	*bla erm bgaB-´recU-pbpA1´*^{Y11A}		This work
pSH507	*bla erm bgaB-´recU-pbpA1´*^{R12A}		This work
pSH508	*bla erm bgaB-´recU-pbpA1´*$^{R8A\ R12A}$		This work
pSH509	*bla erm bgaB-´recU-pbpA1´*^{Q10P}		This work

Supplementary Table 5: Oligonucleotide primers used in this study for BACTH

Name	Sequence, 5' – 3'	Template
***B. subtilis* BACTH**		
**Construction of T25-fusions to *B. subtilis ypbE***		
ypbE-F	GGTATCTAGAGACGAACATGTCGAGAGTAGAGAG	168
ypbE-R	CTAGGTACCTACCCTATTCATCCATTAAAGG	
**Construction of T25-fusions to *B. subtilis rodZ***		
rodZ-F	TGATCTAGAGTCATTGGATGATCTCCAAGCGGC	168
rodZ-R	TGAGGTACCACTTCATAAGTCGTTAACGATCC	
**Construction of T25-fusions to *B. subtilis yrrS***		
yrrS-F	ATGTCTAGAGAGAGCAATTAATCAATCTCGTTATGAAAATCG	168
yrrS-R	ATCGGTACCTGTTTATTTTAGCTTTTCTACTTTTGTCGGCTTCAAG	
**Construction of T25-fusions to *B. subtilis yrrR***		
yrrR-F	GACTCTAGAGAAGATATCGAAACGAATGAAGC	168
yrrR-R	TTAGGTACCTTATTTCTGATTGATTTCAATTTCGTGTAC	
***L. monoctogenes* BACTH**		
**Construction of T25-fusions to *L. monocytogenes sepF***		
JR334	GCGCTCTAGAAGGACTATCGAATAAATTTAAGTCATTC	EGD-e
JR335	GCGCGGTACCGCCATAAAGTTTTGTTCATCGAGCATTTC	
**Construction of T25-fusions to *L. monocytogenes zapA***		
JR337	GCGCCTGCAGCAAATGAGAGAAATAAAGTAGTGAC	EGD-e
JR338	GCGCGGTACCGCATCTCTTCCTTTAATTCGAGCCAG	
**Construction of T25-fusions to *L. monocytogenes ezrA***		
JR339	GCGCTCTAGAATACTACATGTTAATCGGCTTTATTATC	EGD-e
JR340	GCGCGGTACCGCTTCAATATCGTCGACGCTTACTTTTG	
**Construction of a T25-fusion to *L. monocytogenes divIB***		
JR353	GCGCTCTAGAAGCTGAAAATAAACGAGTAATTTCCATTG	EGD-e
JR354	GCGCGGTACCGCTTCATTTGTTTCTTTCTTCTCTTTAGC	
**Construction of a T25-fusion to *L. monocytogenes divIC***		
JR355	GCGCTCTAGAAAAAAAAAGCCAAATCAAAAGTGGCGAG	EGD-e
JR356	GCGCGGTACCGCCTCTTTTTGTTTCGAATTCTCTTCTG	
**Construction of T25-fusions to *L. monocytogenes mreC***		
JR364	GCGCCTGCAGCGCCACAATTTTTTCTCAATAAACGTTTG	EGD-e
JR365	GCGCGGTACCGCTTGGCCTCCAGTCGTGTCTG	
**Construction of T25-fusions to *L. monocytogenes mreBH***		
JR368	GCGCTCTAGAATTTGGAACAACAACTATTGGAATTG	EGD-e
JR369	GCGCGGTACCGCATTTTTAGCTAATTTATCTGCAAAAACG	
**Construction of a T25-fusion to *L. monocytogenes pbpA2***		
SHW153	GACTCTAGAGGACAAATTCAAACAGCAACTTATT	EGD-e

SHW154	CTTAGGTACCTTACCTATCGAATCGATTAAGTTTC	
**Construction of a T25-fusion to *L. monocytogenes pbpB2***		
SHW155	AACTGCAGTGAAACGGCGTATAGGTAACATG	EGD-e
SHW156	CGCGGATCCTTGAGCAAATCACCGATACCG	
**Construction of a T25-fusion to *L. monocytogenes pbpB1***		
SHW157	TGAAACTAAATTTTAGAAAAAAGAA	EGD-e
SHW158	CCGGAATTCTTAATTTTCGGTTTGTTCTGATTG	
**Construction of a T25-fusion to *L. monocytogenes pbpB3***		
SHW159	AACTGCAGTGGCTAGTTATGGTGGGAAAAAG	EGD-e
SHW160	CGCGGATCCTTATACATACTTTCAATTACAGG	
***S. pneumoniae* BACTH**		
**Construction of a T25-fusion to *S. pneumoniae gpsB***		
*gpsB*_PF	AACTGCAGGATGGCAAGTATTATTTTTTCAGCG	D39 and mutants
*gpsB*_BR	CGGGATCCTCAAAATCTGAGTTATCTAAAATTTG	
**Construction of a T25-fusion to *S. pneumoniae mreC***		
*mreC*_XF	GCTCTAGAGATGAACCGTTTTAAAAAATCAAAAT	D39
*mreC*_BR	CGGGATCCTTATGAATTCCCCACTAATTCTATC	
**Construction of a T25-fusion to *S. pneumoniae pbp2a***		
*pbp2a*_XF	CGTCTAGATATGAAATTAGATAAATTATTTGAGAAATTTCTTTCTCTTTTTAAAAAAGAAACAAG	D39 and mutants
*pbp2a*_BR	CGGGATCCTTAGCGAAATAGATTGACTATCGAATCCC	
**Construction of a T25-fusion to *S. pneumoniae pbp1a***		
*pbp1a*_XF	GCTCTAGAGATGAACAAACCAACGATTCTGCG	D39
*pbp1a*_BR	CGGGATCCTTATGGTTGTGCTGGTTGAGGAT	
**Construction of a T25-fusion to *S. pneumoniae pbp2x***		
*pbp2x*_XF	CGTCTAGAGATGAAGTGGACAAAAAGAGTAATCC	D39
*pbp2x*_BR	CGGAATTCTTAGTCTCCTAAAGTTAATGTAAT	
**Construction of a T25-fusion to *S. pneumoniae pbp2b***		
*pbp2b*_XF	GCGGATCCCATGAGACTGATTTGTATGAG	D39
*pbp2b*_BR	CGGAATTCCTAATTCATTGGATGGTATTTTTG	
**Sequencing and verification of *S. pneumoniae* BACTH**		
pKT25_579F	GTTCGCCATTATGCCGCATC	
pKT25_802R	GGATGTGCTGCAAGGCGATT	
pUT18C_484F	GATGTACTGGAAACGGTGC	
pUT18C_660R	CTTAACTATGCGGCATCAGAGC	
pKNT25/pUT18_49F	CGCAATTAATGTGAGTTAGC	
pKNT25_328R	TTGATGCCATCGAGTACG	
pUT18_304R	CGAGCGATTTTCCACAACAA	
*pbp2a*_1010F	AGAGCTGGACCAAAACTACC	
*pbp2a*_1106R	CGGTTCGAGAGCTACACTTC	

*pbp1a*_980F	GCAAGTCGCTTCTACCATTG
*pbp1a*_1126R	CAAGGCAGGAGCATAGTCTG
*pbp2x*_1055F	CCTTTCCAGGAGGAGAAGTC
*pbp2x*_1177R	CCAACGTTACTTGAGTGTGC
*pbp2b*_943F	GGCTTTCCAAGATAGCGTGG
*pbp2b*_1047R	AAACCGCACCTGTTTTTGGG

Supplementary Table 6: Oligonucleotide primers used in this study for *L. monocytogenes* and *S. pneumoniae* strain construction

***L. monoctogenes* strain construction**			
**Introduction of T7A into *pbpA1***			
SHW744	CCGCAGGCAAGATCTCAGTATCGCAATAAAC	pSH437, pSH497	$pbpA1^{T7A}$
SHW754	AGATCTTGCCTGCGGTTTATCTGCCTCTAG		
SHW745	AGATCTTGCCTGCGGTTTATCTGCCAT		
**Introduction of R8A into *pbpA1***			
SHW746	CAGACAGCATCTCAGTATCGCAATAAACAAAG	pSH437, pSH497	$pbpA1^{R8A}$
SHW747	CTGAGATGCTGTCTGCGGTTTATCTGCC		
**Introduction of R12A into *pbpA1***			
SHW748	CAGTATGCAAATAAACAAAGTGGTGGTTCTAAA	pSH437, pSH497	$pbpA1^{R12A}$
SHW749	TTTATTTGCATACTGAGATCTTGTCTGCGG		
**Introduction of R8A R12A into *pbpA1***			
SHW750	CAGACAGCATCTCAGTATGCAAATAAACAAAGTGGTGGTTCTAAA	pSH497	$pbpA1^{R8AR12A}$
SHW751	GTTTATTTGCATACTGAGATGCTGTCTGCGGTTTATCTGCC		
**Deletion of GpsB N-terminus**			
SHW752	TCGACTCTAGAGCCGCTTAGAACTTCAACACAACC	pSH226	$gpsB_{\Delta N}$
SHW753	TAAGCGGCTCTAGAGTCGACCTGCAGG		
**Introduction of Y11A into *pbpA1***			
SHW755	TCTCAGGCTCGCAATAAACAAAGTGGTGG	pSH437	$pbpA1^{Y11A}$
SHW756	ATTGCGAGCCTGAGATCTTGTCTGCGG		
**Introduction of K14A into *pbpA1***			
SHW757	CGCAATGCACAAAGTGGTGGTTCTAAAAAG	pSH437	$pbpA1^{K14A}$
SHW758	ACTTTGTGCATTGCGATACTGAGATCTTGTC		
**Introduction of K20A into *pbpA1***			
SHW759	GGTTCTGCAAAGAAATCCCAAAAACGAGG	pSH437	$pbpA1^{K20A}$
SHW760	TTTCTTTGCAGAACCACCACTTTGTTTATTG		
**Introduction of K21A into *pbpA1***			
SHW761	TCTAAAGCGAAATCCCAAAAACGAGGAAAAC	pSH437	$pbpA1^{K21A}$
SHW762	GGATTTCGCTTTAGAACCACCACTTTGTTTATTG		
**Introduction of K22A into *pbpA1***			
SHW763	AAAAAGGCATCCCAAAAACGAGGAAAACG	pSH437	$pbpA1^{K22A}$
SHW764	TTGGGATGCCTTTTTAGAACCACCACTTTG		
**Introduction of K25A into *pbpA1***			
SHW765	TCCCAAGCACGAGGAAAACGAGTAGCAG	pSH437	$pbpA1^{K25A}$
SHW766	TCCTCGTGCTTGGGATTTCTTTTTAGAACCAC		
**Introduction of R26A into *pbpA1***			
SHW767	CAAAAAGCAGGAAAACGAGTAGCAGCG	pSH437	$pbpA1^{R26A}$
SHW768	TTTTCCTGCTTTTTGGGATTTCTTTTTAGAACC		

**Introduction of K28A into *pbpA1***			
SHW769	CGAGGAGCACGAGTAGCAGCGAATATTTTC	pSH437	*pbpA1*^{K28A}
SHW770	TACTCGTGCTCCTCGTTTTTGGGATTTCTTTTTAG		
**Introduction of R29A into *pbpA1***			
SHW771	GGAAAAGCAGTAGCAGCGAATATTTTCAAAAC	pSH437	*pbpA1*^{R29A}
SHW772	TGCTACTGCTTTTCCTCGTTTTTGGGATTTCTT		
**Construction of pMAD-*pbpA1´* (pSH497)**			
SHW773	GCGCGCGGATCCCGAAGGTACGTTCTATTTATGAG	EGD-e	*pbpA1´*
SHW774	GCGCGCCCATGGGTTGGAGCGGTTCCGGATAAG		
**Deletion of *pbpA1* N-terminus**			
SHW775	TATTTAGTCGACCATTAAAAATCTCTCTCCTTTAAA	pSH497	*pbpA1´*$_{\Delta N}$
SHW776	TTAATGGTCGACTAAATATACGTATTCTGGTGGAACCCC		
**Correction of pMAD-*pbpA1´* (pSH497)**			
SHW777	GATATCGGATCCATTGGTTACCCTAACGGCAAGAAG	pSH497	*pbpA1´*
SHW778	ACCAATGGATCCGATATCGCCCGACGCGAGG		
**Introduction of Q10P into *pbpA1***			
SHW787	AGATCTCCGTATCGCAATAAACAAAGTGGTGG	pSH497	*pbpA1*^{Q10P}
SHW788	GCGATACGGAGATCTTGTCTGCGGTTTATC		
***S. pneumoniae* strain construction**			
**Construction of IU8496 (Δ*divIVA*::P_c-*erm*)**			
TT242	GGGAATGGAATGGATAAAGAAGGTAGAAGA	D39	Upstream of *divIVA* to 8 bp before *divIVA* ORF
SC216	CATTATCCATTAAAAATCAAACGGATCCTTACTTACTTAATAATAACTGGACGGTTA		
SC215	TAACCGTCCAGTTATTATTAAGTAAGTAAGGATCCGTTTGATTTTTAATGGATAATGTG	E177	P_c-*erm*
SC218	GCTGGTGTTGGACCTGTCGGATGCACTGGAGGGGCCCCTTTCCTTATGCTTTTGGAC		
SC217	GTCCAAAAGCATAAGGAAAGGGGCCCCTCCAGTGCATCCGACAGGTCCAACACCAGC	D39	Downstream of *divIVA*
TT238	TTCAGCAAGGGCTGACTCAGATGACCATGA		
**Construction of IU11051 (*gpsB*$^{+}$-P_c-*erm*)**			
TT196	GCCAAGCCCTGAGACAAATAGTAGTCGTTGGT	D39	Upstream of *gpsB* + *gpsB*$^{+}$
TT905	ACAAATTTTGGGCCCGGTTAAAAATCTGAGTTATCTAAAATTTGTTTACCAAA		
TT906	GTAAACAAATTTTAGATAACTCAGATTTTTAACCGGGCCCAAAATTTGTTTGAT	IU5838	P_c-*erm* + downstream of *gpsB*
TT197	TTTGATACGATCTGCTGCCCGAAGCCAAAGGT		
**Construction of IU11286 (Δ*bgaA*::*tet*-P_{Zn}-RBS^{ftsA}-*gpsB*$^{+}$)**			
TT657	CGCCCCAAGTTCATCACCAATGACATCAAC	IU8122	5’ fragment Δ*bgaA*::*tet*-P_{zn} RBS^{ftsA}
JC03	CTTTCGCTGAAAAAATAATACTTGCCATTACATCGCTTCCTCTCTATCTTCCTTGTTATA		
JC04	GGAAGATAGAGAGGAAGCGATGTAATGGCAAGTATTATTTTTTCAGCGAAAG	D39	middle fragment *gpsB*$^{+}$
JC05	GTTTATGAGAAAGTAAGTTCTTTTAAAAATCTGAGTTATCTAAAATTTGTTTACCAAAAA		
JC06	AAACAAATTTTAGATAACTCAGATTTTTAAAAGAACTTACTTTCTCATAAACCAGTTGCT	IU8122	3’ *bgaA* fragment
CS121	GCTTTCTTGAGGCAATTCACTTGGTGC		
**Construction of IU12361 (*gpsB*^{D29A}-P_c-*erm*)**			
TT196	GCCAAGCCCTGAGACAAATAGTAGTCGTTGGT	D39	Upstream and 5’ *gpsB*^{D29A}

JM072	ATGACATCGTCTAAAAACTCGGCAACTTCTACTTTATTATAGCC		
JM073	GGCTATAATAAAGTAGAAGTTGCCGAGTTTTTAGACGATGTCAT	IU11051	3’*gpsB*^{D29A}-P$_c$-*erm* + downstream
TT197	TTTGATACGATCTGCTGCCCGAAGCCAAAGGT		
**Construction of IU12363 (*gpsB*^{D33A}-P$_c$-*erm*)**			
TT196	GCCAAGCCCTGAGACAAATAGTAGTCGTTGGT	D39	Upstream and 5’ *gpsB*^{D33A}
JM074	TCCTTGATGACATCGGCTAAAAACTCGTCAACTTCTA		
JM075	TAGAAGTTGACGAGTTTTTAGCCGATGTCATCAAGGA	IU11051	3’*gpsB*^{D33A}-P$_c$-*erm* + downstream
TT197	TTTGATACGATCTGCTGCCCGAAGCCAAAGGT		
**Construction of IU12440 (*gpsB*^{Y23A}-P$_c$-*erm*)**			
TT196	GCCAAGCCCTGAGACAAATAGTAGTCGTTGGT	D39	Upstream and 5’ *gpsB*^{Y23A}
JM070	CAACTTCTACTTTATTAGCGCCACGGACTTCACG		
JM071	CGTGAAGTCCGTGGCGCTAATAAAGTAGAAGTTG	IU11051	3’*gpsB*^{Y23A}-P$_c$-*erm* + downstream
TT197	TTTGATACGATCTGCTGCCCGAAGCCAAAGGT		
**Construction of IU12612 (*gpsB*^{V28A}-P$_c$-*erm*)**			
TT196	GCCAAGCCCTGAGACAAATAGTAGTCGTTGGT	D39	Upstream and 5’ *gpsB*^{V28A}
JM091	CATCGTCTAAAAACTCGTCAGCTTCTACTTTATTATAGCC		
JM092	GGCTATAATAAAGTAGAAGCTGACGAGTTTTTAGACGATG	IU11051	3’*gpsB*^{V28A}-P$_c$-*erm* + downstream
TT197	TTTGATACGATCTGCTGCCCGAAGCCAAAGGT		
**Construction of IU12615 (*gpsB*^{L32A}-P$_c$-*erm*)**			
TT196	GCCAAGCCCTGAGACAAATAGTAGTCGTTGGT	D39	Upstream and 5’ *gpsB*^{L32A}
JM093	CTTGATGACATCGTCTGCAAACTCGTCAACTTCTAC		
JM094	GTAGAAGTTGACGAGTTTGCAGACGATGTCATCAAG	IU11051	3’*gpsB*^{L32A}-P$_c$-*erm* + downstream
TT197	TTTGATACGATCTGCTGCCCGAAGCCAAAGGT		
**Construction of IU13121 (*gpsB*^{I36A}-P$_c$-*erm*)**			
TT196	GCCAAGCCCTGAGACAAATAGTAGTCGTTGGT	D39	Upstream and 5’ *gpsB*^{I36A}
JM076	CATAGGTTTCATAGTCCTTGGCGACATCGTCTAAAAAC		
JM077	GTTTTTAGACGATGTCGCCAAGGACTATGAAACCTATG	IU11051	3’*gpsB*^{I36A}-P$_c$-*erm* + downstream
TT197	TTTGATACGATCTGCTGCCCGAAGCCAAAGGT		
**Construction of IU13180 (*pbp2a*$_{\Delta32\text{-}37}$)**			
P226	GGTACGACAACGAAATGTCATACACTGCAC	D39	Upstream and 5’ *pbp2a*$_{\Delta32\text{-}37}$
JM057	TTTTCGAATCGGACCTACTTGGGCTAATTTGCGACGTAAGATAGTAGAATCAGAGTCCTC		
JM058	GAGGACTCTGATTCTACTATCTTACGTCGCAAATTAGCCCAAGTAGGTCCGATTCGAAAA	D39	*3’ pbp2a*$_{\Delta32\text{-}37}$ + downstream
P227	TCTGTTCCCGTGTGATCCGACAAATCCT		
**Construction of IU13256 (Δ*pbp2a* markerless, Δ31-712)**			
P226	GGTACGACAACGAAATGTCATACACTGCAC	D39	Upstream and 5’ 90 bp of *pbp2a*
TT1013	TTTGAGCCTTTTCCTTAATCTTCGCACGTAAGATAGTAGAATCAGAGTCCTCTAGTTCAC		
TT1014	AACTAGAGGACTCTGATTCTACTATCTTACGTGCGAAGATTAAGGAAAAGGCTCAAACAA	D39	*3’* 60 *bp* of *pbp2a* + downstream
P227	TCTGTTCCCGTGTGATCCGACAAATCCT		
**Construction of IU13258 (*pbp2a*$_{\Delta2\text{-}49}$)**			
P226	GGTACGACAACGAAATGTCATACACTGCAC	D39	Upstream and 5’ *pbp2a*$_{\Delta2\text{-}49}$

TT1015	AGTATAAGGATAATCTTTGTTAGATGATACATGCGTTTATTTTATCATCTTCATCATAGG		
TT1016	AAGATGATAAAATAAACGCATGTATCATCTAACAAAGATTATCCTTATACTAGGTTTGAG	D39	*3' pbp2a*$_{\Delta 2\text{-}49}$ + downstream
P227	TCTGTTCCCGTGTGATCCGACAAATCCT		
**Construction of IU13298 (*pbp2a*$_{\Delta 27\text{-}38}$)**			
P226	GGTACGACAACGAAATGTCATACACTGCAC	D39	Upstream and 5' *pbp2a*$_{\Delta 27\text{-}38}$
TT1020	AATCGGACCTACTTGGGCTAAAGAATCAGAGTCCTCTAGTTCACTTGTTTCTT		
TT1021	AACAAGTGAACTAGAGGACTCTGATTCTTTAGCCCAAGTAGGTCCGATTCGA	D39	*3' pbp2a*$_{\Delta 27\text{-}38}$ + downstream
P227	TCTGTTCCCGTGTGATCCGACAAATCCT		
**Construction of IU13301 (*pbp2a*$_{\Delta 26\text{-}45}$)**			
P226	GGTACGACAACGAAATGTCATACACTGCAC	D39	Upstream and 5' *pbp2a*$_{\Delta 26\text{-}45}$
TT1022	TAACGACGCCAGAATTTTCGATCAGAGTCCTCTAGTTCACTTGTTTCTTTTT		
TT1023	AGAAACAAGTGAACTAGAGGACTCTGATCGAAAATTCTGGCGTCGTTATCAT	D39	*3' pbp2a*$_{\Delta 26\text{-}45}$ + downstream
P227	TCTGTTCCCGTGTGATCCGACAAATCCT		
**Construction of IU13364 (*gpsB*^{Y23A}-FLAG-P$_c$-*erm*)**			
AL298	GAGGGAAGGCACCAGCCTTGATTTCA	IU12440	Upstream and *gpsB*^{Y23A}
TT262	TTATTTATCATCATCATCTTTATAATCAAAATCTGAGTTATCTAAAATTTGTTTACCAAA		
TT263	TAACTCAGATTTTGATTATAAAGATGATGATGATAAATAACCGGGCCCAAAATTTGTTTG	IU5838	FLAG-P$_c$-*erm* + downstream
TT197	TTTGATACGATCTGCTGCCCGAAGCCAAAGGT		
**Construction of IU13366 (*gpsB*^{V28A}-FLAG-P$_c$-*erm*)**			
AL298	GAGGGAAGGCACCAGCCTTGATTTCA	IU12612	Upstream and *gpsB*^{V28A}
TT262	TTATTTATCATCATCATCTTTATAATCAAAATCTGAGTTATCTAAAATTTGTTTACCAAA		
TT263	TAACTCAGATTTTGATTATAAAGATGATGATGATAAATAACCGGGCCCAAAATTTGTTTG	IU5838	FLAG-P$_c$-*erm* + downstream
TT197	TTTGATACGATCTGCTGCCCGAAGCCAAAGGT		
**Construction of IU13368 (*gpsB*^{D29A}-FLAG-P$_c$-*erm*)**			
AL298	GAGGGAAGGCACCAGCCTTGATTTCA	IU13141	Upstream and *gpsB*^{D29A}
TT262	TTATTTATCATCATCATCTTTATAATCAAAATCTGAGTTATCTAAAATTTGTTTACCAAA		
TT263	TAACTCAGATTTTGATTATAAAGATGATGATGATAAATAACCGGGCCCAAAATTTGTTTG	IU5838	FLAG-P$_c$-*erm* + downstream
TT197	TTTGATACGATCTGCTGCCCGAAGCCAAAGGT		
**Construction of IU13370 (*gpsB*^{L32A}-FLAG-P$_c$-*erm*)**			
AL298	GAGGGAAGGCACCAGCCTTGATTTCA	IU12615	Upstream and *gpsB*^{L32A}
TT262	TTATTTATCATCATCATCTTTATAATCAAAATCTGAGTTATCTAAAATTTGTTTACCAAA		
TT263	TAACTCAGATTTTGATTATAAAGATGATGATGATAAATAACCGGGCCCAAAATTTGTTTG	IU5838	FLAG-P$_c$-*erm* + downstream
TT197	TTTGATACGATCTGCTGCCCGAAGCCAAAGGT		
**Construction of IU13372 (*gpsB*^{D33A}-FLAG-P$_c$-*erm*)**			
AL298	GAGGGAAGGCACCAGCCTTGATTTCA	IU12363	Upstream and *gpsB*^{D33A}
TT262	TTATTTATCATCATCATCTTTATAATCAAAATCTGAGTTATCTAAAATTTGTTTACCAAA		
TT263	TAACTCAGATTTTGATTATAAAGATGATGATGATAAATAACCGGGCCCAAAATTTGTTTG	IU5838	FLAG-P$_c$-*erm* + downstream
TT197	TTTGATACGATCTGCTGCCCGAAGCCAAAGGT		
**Construction of IU13374 (*gpsB*^{I36A}-FLAG-P$_c$-*erm*)**			
AL298	GAGGGAAGGCACCAGCCTTGATTTCA	IU13121	Upstream and *gpsB*^{Y23A}

TT262	TTATTTATCATCATCATCTTTATAATCAAAATCTGAGTTATCTAAAATTTGTTTACCAAA		
TT263	TAACTCAGATTTTGATTATAAAGATGATGATGATAAATAACCGGGCCCAAAATTTGTTTG	IU5838	FLAG-P_c-*erm* + downstream
TT197	TTTGATACGATCTGCTGCCCGAAGCCAAAGGT		
**Construction of IU14256 (*pbp2a*^{R31A})**			
P226	GGTACGACAACGAAATGTCATACACTGCAC	D39	Upstream and 5’ *pbp2a*^{R31A}
TT1056	TTTCGATCACTACGAGAGGCACGTAAGATAGTAGAATCAG		
TT1057	CTGATTCTACTATCTTACGTGCCTCTCGTAGTGATCGAAA	D39	*3’ pbp2a*^{R31A} + downstream
P227	TCTGTTCCCGTGTGATCCGACAAATCCT		
**Construction of IU14259 (*pbp2a*$^{R31K\ R33K}$)**			
P226	GGTACGACAACGAAATGTCATACACTGCAC	D39	Upstream and 5’ *pbp2a*$^{R31K\ R33K}$
TT1058	CTAATTTTTTTCGATCACTCTTAGATTTACGTAAGATAGTAGAATCAG		
TT1059	CTGATTCTACTATCTTACGTAAATCTAAGAGTGATCGAAAAAAATTAG	D39	*3’ pbp2a*$^{R31K\ R33K}$ + downstream
P227	TCTGTTCCCGTGTGATCCGACAAATCCT		
**Construction of IU14263 (*pbp2a*^{R33A})**			
P226	GGTACGACAACGAAATGTCATACACTGCAC	D39	Upstream and 5’ *pbp2a*^{R33A}
JM047	TTTTCGATCACTGGCAGAGCGACGTAAGAT		
JM048	ATCTTACGTCGCTCTGCCAGTGATCGAAAA	D39	*3’ pbp2a*^{R33A} + downstream
P227	TCTGTTCCCGTGTGATCCGACAAATCCT		
**Construction of IU14318 (Δ*bgaA*::*kan*-P_{Zn}-RBS^{ftsA}-*pbp2a*$^{+}$)**			
P146	TGGCCATTCATCGCTGGTCGTGCTGAAAT	IU12788	5’ fragment Δ*bgaA*::*kan*-P_{zn}-RBS^{ftsA}
JC07	ATTTCTCAAATAATTTATCTAATTTCATTACATCGCTTCCTCTCTATCTTCCTTGTTATA		
JC08	GAAGATAGAGAGGAAGCGATGTAATGAAATTAGATAAATTATTTGAGAAATTTCTTTCTC	D39	middle fragment *pbp2a*$^{+}$
JC09	ACTGGTTTATGAGAAAGTAAGTTCTTTTATTAGCGAAATAGATTGACTATCGAATCCC		
JC10	ATTCGATAGTCAATCTATTTCGCTAATAAAAGAACTTACTTTCTCATAAACCAGTTGCT	D39	3’ *bgaA* fragment
CS121	GCTTTCTTGAGGCAATTCACTTGGTGC		
**Construction of IU14394 (*pbp2a*$_{\Delta 29\text{-}36}$)**			
P226	GGTACGACAACGAAATGTCATACACTGCAC	D39	Upstream and 5’ *pbp2a*$_{\Delta 29\text{-}36}$
TT1091	CGGACCTACTTGGGCTAATTTTTTGATAGTAGAATCAGAGTCCTCTAGTTCACTTGTTTC		
TT1092	AGTGAACTAGAGGACTCTGATTCTACTATCAAAAAATTAGCCCAAGTAGGTCCGATT	D39	*3’ pbp2a*$_{\Delta 29\text{-}36}$ + downstream
P227	TCTGTTCCCGTGTGATCCGACAAATCCT		
**Construction of IU14396 (*pbp2a*$_{\Delta 31\text{-}36}$)**			
P226	GGTACGACAACGAAATGTCATACACTGCAC	D39	Upstream and 5’ *pbp2a*$_{\Delta 31\text{-}36}$
TT1093	GGACCTACTTGGGCTAATTTTTTACGTAAGATAGTAGAATCAGAGTCCTCTAGTTCAC		
TT1094	CTAGAGGACTCTGATTCTACTATCTTACGTAAAAAATTAGCCCAAGTAGGTCCGATT	D39	*3’ pbp2a*$_{\Delta 31\text{-}36}$ + downstream
P227	TCTGTTCCCGTGTGATCCGACAAATCCT		
**Construction of IU14400 (*pbp2a*$^{R31A\ S32A\ R36A}$)**			
P226	GGTACGACAACGAAATGTCATACACTGCAC	D39	Upstream and 5’ *pbp2a*$^{R31A\ S32A\ R36A}$
TT1095	ATTTTTTTGCATCACTACGAGCGGCACGTAAGATAGTAGAATCAGAGTCCTCTAGTTCAC		
TT1096	TCTACTATCTTACGTGCCGCTCGTAGTGATGCAAAAAAATTAGCCCAAGTAGGTCCGATT	D39	*3’ pbp2a*$^{R31A\ S32A\ R36A}$ + downstream
P227	TCTGTTCCCGTGTGATCCGACAAATCCT		

**Construction of IU14502 (*pbp2a*$_{\Delta 2\text{-}22}$)**			
P226	GGTACGACAACGAAATGTCATACACTGCAC	D39	Upstream and 5’ *pbp2a*$_{\Delta 2\text{-}22}$
TT1097	GCGACGTAAGATAGTAGAATCAGAGTCCATGCGTTTATTTTATCATCTTCATCATAGG		
TT1098	GATGAAGATGATAAAATAAACGCATGGACTCTGATTCTACTATCTTACGTCGCTCTC	D39	*3’ pbp2a*$_{\Delta 2\text{-}22}$ + downstream
P227	TCTGTTCCCGTGTGATCCGACAAATCCT		

Supplementary Table 7: Oligonucleotide primers used in this study for recombinant protein production

**Name**	**Sequence, 5' – 3'**
**Construction of plasmid expressing recombinant *Bs*GpsB$_{5-64}$**	
BsGpsB5start	GATGCTTCATATGAAAGTAAAGCTTTCTGCGAAAGAAATTTTGG
BsGpsB64stop	CTGTTTCTCGAGGGCTTCTTCAAGCTGTTTTTCAGCTG
**Introduction of E17A to *Bs*GpsB$_{1-63}$**	
BsGpsBE17A5	CTGCGAAAGAAATTTTGGAAAAAGCATTTAAAACAGGCGTTAGAGGC
BsGpsBE17A3	GCCTCTAACGCCTGTTTTAAATGCTTTTTCCAAAATTTCTTTCGCAG
**Introduction of Y25F to *Bs*GpsB$_{1-63}$**	
BsGpsBY25F5	CAGGCGTTAGAGGCTTCAAGCAAGAAGACGTTGAC
BsGpsBY25F5	GTCAACGTCTTCTTGCTTGAAGCCTCTAACGCCTG
**Introduction of D31A to *Bs*GpsB$_{1-63}$**	
BsGpsBD31A5	CGTTAGAGGCTACAAGCAAGAAGACGTTGCCAAATTTTTAGATATGATTATTAAGG
BsGpsBD31A3	CCTTAATAATCATATCTAAAAATTTGGCAACGTCTTCTTGCTTGTAGCCTCTAACG
**Introduction of D35A to *Bs*GpsB$_{1-63}$**	
BsGpsBD35A5	GAAGACGTTGACAAATTTTTAGCTATGATTATTAAGGATTATGAAACCTTCCATC
BsGpsBD35A3	GATGGAAGGTTTCATAATCCTTAATAATCATAGCTAAAAATTTGTCAACGTCTTC
**Introduction of S16C to *Bs*PBP1$_{1-17}$**	
BsPBP1S16C5	GCTCGACGAAAAGCGAACTGCAAATCGAGTCCTTCACC
BsPBP1S16C3	GGTGAAGGACTCGATTTGCAGTTCGCTTTTCGTCGAGC
**Introduction of S7A to *Bs*PBP1$_{1-17}$**	
BsPBP1S7A5	CCATGGCAGATCAATTTAACGCCCGTGAAGCTCGACGAAAAGC
BsPBP1S7A3	GCTTTTCGTCGAGCTTCACGGGCGTTAAATTGATCTGCCATGG
**Introduction of R8A to *Bs*PBP1$_{1-17}$**	
BsPBP1R8A5	GCAGATCAATTTAACAGCGCTGAAGCTCGACGAAAAGCGAACTG
BsPBP1R8A3	CAGTTCGCTTTTCGTCGAGCTTCAGCGCTGTTAAATTGATCTGC
**Introduction of A10P to *Bs*PBP1$_{1-17}$**	
BsPBP1A10P5	GATCAATTTAACAGCCGTGAACCTCGACGAAAAGCGAACTGC
BsPBP1A10P3	GCAGTTCGCTTTTCGTCGAGGTTCACGGCTGTTAAATTGATC
**Introduction of R11A to *Bs*PBP1$_{1-17}$**	
BsPBP1R11A5	CAATTTAACAGCCGTGAAGCTGCACGAAAAGCGAACTGCAAATCG
BsPBP1R11A3	CGATTTGCAGTTCGCTTTTCGTGCAGCTTCACGGCTGTTAAATTG
**Introduction of R28A to *Bs*PBP1$_{1-32}$**	
BsPBP1R28A5	CACCGAAAAAAGGCAAGAAAGCAAAAAAGGGCGGATAGTTTAAAAAG
BsPBP1R28A3	CTTTTTAAACTATCCGCCCTTTTTTGCTTTCTTGCCTTTTTTCGGTG
**Introduction of R8K to *Bs*PBP1$_{1-17}$**	
BsPBP1AR8K5	CATGGCAGATCAATTTAACAGCAAAGAAGCTCGACGAAAAGCGAACTGC
BsPBP1AR8K3	GCAGTTCGCTTTTCGTCGAGCTTCTTTGCTGTTAAATTGATCTGCCATG
**Introduction of R11K to *Bs*PBP1$_{1-17}$**	
BsPBP1AR11K5	CAGATCAATTTAACAGCCGTGAAGCTAAACGAAAAGCGAACTGCAAATCG
BsPBP1AR11K3	CGATTTGCAGTTCGCTTTTCGTTTAGCTTCACGGCTGTTAAATTGATCTG
**Construction of plasmid expressing recombinant MBP-*Lm*PBPA1$_{1-20}$**	
LmPBPA1ncoI5	GATTTTCCATGGCAGATAAACCGCAGACAAG
LmPBPA1xhoI3	GTAGACCCTCGAGAACGTTTTACTTGGGTTGCATAGTTATAAC
**Introduction of S19C, K21STOP to MBP-*Lm*PBPA1$_{1-20}$ to generate MBP-*Lm*PBPA1$_{1-20}^{S19C}$**	
LmPBPA1S19CK21STOP5	CAGTATCGCAATAAACAAAGTGGTGGTTGTAAATAGAAATCCCAAAAACGAGG
LmPBPA1S19CK21STOP3	CCTCGTTTTTGGGATTTCTATTTACAACCACCACTTTGTTTATTGCGATACTG
**Introduction of R8A to *Lm*PBPA1$_{1-20}^{S19C}$**	
LmPBPA1R8A5	GGCAGATAAACCGCAGACAGCATCTCAGTATCGCAATAAACAAAG
LmPBPA1R8A3	CTTTGTTTATTGCGATACTGAGATGCTGTCTGCGGTTTATCTGCC
**Introduction of Q10P to *Lm*PBPA1$_{1-20}^{S19C}$**	
LmPBPA1Q10P5	GATAAACCGCAGACAAGATCTCCGTATCGCAATAAACAAAGTGG
LmPBPA1Q10P3	CCACTTTGTTTATTGCGATACGGAGATCTTGTCTGCGGTTTATC

**Introduction of Y11A to *Lm*PBPA1$_{1-20}$^{S19C}**	
LmPBPA1Y11A5	GGCAGATAAACCGCAGACAAGATCTCAGGCTCGCAATAAACAAAGTGGTGG TTG
LmPBPA1Y11A3	CAACCACCACTTTGTTTATTGCGAGCCTGAGATCTTGTCTGCGGTTTATCTGC C
**Introduction of R12A to *Lm*PBPA1$_{1-20}$^{S19C}**	
LmPBPA1R12A5	GATAAACCGCAGACAAGATCTCAGTATGCCAATAAACAAAGTGGTGGTTG
LmPBPA1R12A3	CAACCACCACTTTGTTTATTGGCATACTGAGATCTTGTCTGCGGTTTATC
**Introduction of S16R to *Lm*PBPA1$_{1-20}$^{S19C}**	
LmPBPA1S16R5	CTCAGTATCGCAATAAACAACGTGGTGGTTGTAAATAGAAATCCC
LmPBPA1S16R3	GGGATTTCTATTTACAACCACCACGTTGTTTATTGCGATACTGAG
**Construction of plasmid expressing recombinant MBP-*Sp*PBP2a$_{23-46}$**	
SpPBP2AncoI5	GAAACAAGTGAACCCATGGACTCTGATTCTACTATCTTACG
SpPBP2AxhoI3	CCGGCCCCTCGAGCATCCCTGCCAGAGTCGCAGC
**Introduction of G43C, R46STOP to MBP-*Sp*PBP2a$_{23-46}$ to generate MBP-*Sp*PBP2a$_{23-45}$^{G43C}**	
SpPBP2AG43CR46STOP5	GTGATCGAAAAAAATTAGCCCAAGTATGTCCGATTTGAAAATTCTGGCGTC GTTATC
SpPBP2AG43CR46STOP3	GATAACGACGCCAGAATTTTCAAATCGGACATACTTGGGCTAATTTTTTTCG ATCAC
**Introduction of R33K to MBP-*Sp*PBP2a$_{23-45}$^{G43C}**	
SpPBP2AR33K5	CTCTGATTCTACTATCTTACGTCGCTCTAAAAGTGATCGAAAAAAATTAGCC CAAG
SpPBP2AR33K3	CTTGGGCTAATTTTTTTCGATCACTTTTAGAGCGACGTAAGATAGTAGAATC AGAG
**Introduction of S32A to MBP-*Sp*PBP2a$_{23-45}$^{G43C}**	
SpPBP2AS32A5	GACTCTGATTCTACTATCTTACGTCGCGCTCGTAGTGATCGAAAAAAATTAG CCC
SpPBP2AS32A3	GGGCTAATTTTTTTCGATCACTACGAGCGCGACGTAAGATAGTAGAATCAG AGTC
**Introduction of R33A to MBP-*Sp*PBP2a$_{23-45}$^{G43C}**	
SpPBP2AR33A5	GACTCTGATTCTACTATCTTACGTCGCTCTGCTAGTGATCGAAAAAAATTAG CCC
SpPBP2AR33A3	GGGCTAATTTTTTTCGATCACTAGCAGAGCGACGTAAGATAGTAGAATCAG AGTC
**Introduction of D35P to MBP-*Sp*PBP2a$_{23-45}$^{G43C}**	
SpPBP2AD35P5	CTATCTTACGTCGCTCTCGTAGTCCTCGAAAAAAATTAGCCCAAGTATGTCC
SpPBP2AD35P3	GGACATACTTGGGCTAATTTTTTTCGAGGACTACGAGAGCGACGTAAGATA G
**Introduction of R31K, R33K to MBP-*Sp*PBP2a$_{23-45}$^{G43C}**	
SpPBP2AR31KR33K5	CAGGGTTCCATGGACTCTGATTCTACTATCTTACGTAAATCTAAAAGTGATC G
SpPBP2AR31KR33K3	CGATCACTTTTAGATTTACGTAAGATAGTAGAATCAGAGTCCATGGAACCCT G
**Introduction of I28A, L29A to MBP-*Sp*PBP2a$_{23-45}$^{G43C}**	
SpPBP2AI28AL29A5	GTTCCATGGACTCTGATTCTACTGCCGCACGTCGCTCTCGTAGTGATCG
SpPBP2AI28AL29A5	CGATCACTACGAGAGCGACGTGCGGCAGTAGAATCAGAGTCCATGGAAC
**Construction of plasmid expressing recombinant *Sp*GpsB$_{1-63}$**	
SpGpsBndeI5	GAGAGACATATGGCAAGTATTATTTTTTCAGC
SpGpsBxhoI3	GCACATCTCGAGTAACTACTTAAAAATCTGAGTTATC
**Introduction of NcoI site to plasmid expressing recombinant *Sp*GpsB$_{1-63}$**	
SpGpsBM11NcoI	GCTATACCATGGCAAGTATTATTTTTTCAGCG
SpGpsBM11XhoI	CGATATCTCGAGTAACTACTTAAAAATCTGAGTTATC
**Deletion of residues 1-3 to generate plasmid expressing recombinant *Sp*GpsB$_{4-63}$**	
SpGpsB1to3del5	CCACTACTGAGAATCTTTATTTTCAGGGCGCCATTATTTTTTCAGCG
SpGpsB1to3del3	CGCTGAAAAAATAATGGCGCCCTGAAAATAAAGATTCTCAGTAGTGG
**Construction of plasmid expressing recombinant *Sp*PBP2x$_{1-29}$**	
SpPBP2x5	GCGGAGTAAGCCATGGAGTGGACAAAAAGAGTAATCCG
SpPBP2x3st1	CGTTACTTGAGTGTGCAAAACCTTGAGAAAAAGTC
SpPBP2x3st2	CGTTACTTGAGTGTGCAAAACCTCGAGAAAAAGTC
SpPBP2xS30CL31SL32ST OP5	GAAAACAGACGCAGAGTTGGAAAAAGTCTGTGTTCATAATCTGTCTTTGTTT TTGCC

SpPBP2xS30CL31SL32STOP3	GGCAAAAACAAAGACAGATTATGAACACAGACTTTTTCCAACTCTGCGTCTGTTTTC
SpPBP2xinsgly5	CCTGTACTTCCAGGGTTCCGGATCTGGAATGGAGTGGACAAAAAGAG
SpPBP2xinsgly3	CTCTTTTTGTCCACTCCATTCCAGATCCGGAACCCTGGAAGTACAGG
**Construction of plasmid expressing recombinant *Bs*YpbE$_{80-240}$**	
YpbEndeI5	CTTATTTCATATGAAGAGCCACCCGGATAATCATG
YpbExhoI3	CGTAAACTCGAGATAATACCCTATTCATCCATTAAAGG
**Construction of plasmid expressing recombinant *Bs*YpbE$_{130-240}$**	
YpbEtruncatencoi5	GCTTCTCCATGGAAGATTCCAAGCCAAAAGAGC
YpbEtruncatencoi3	GCTGCTGCCCATGGTATATCTC
**Construction of plasmid expressing recombinant *Bs*YrrS**	
YrrSndei5	GCAGAACATATGAGCAATAATCAATCTCGTTATG
YrrSxhoi3	CGGCTGCTCGAGTTTATTTTAGCTTTTCTACTTTTGTC
**Construction of plasmid expressing recombinant *Bs*YrrSΔ$_{13-16}$**	
YrrS$_{13-16}$delete5	CTCGTTATGAAAATCGTGATGCCAATTTAGTGCTTAACATTTTAATCG
YrrS$_{13-16}$delete3	CGATTAAAATGTTAAGCACTAAATTGGCATCACGATTTTCATAACGAG

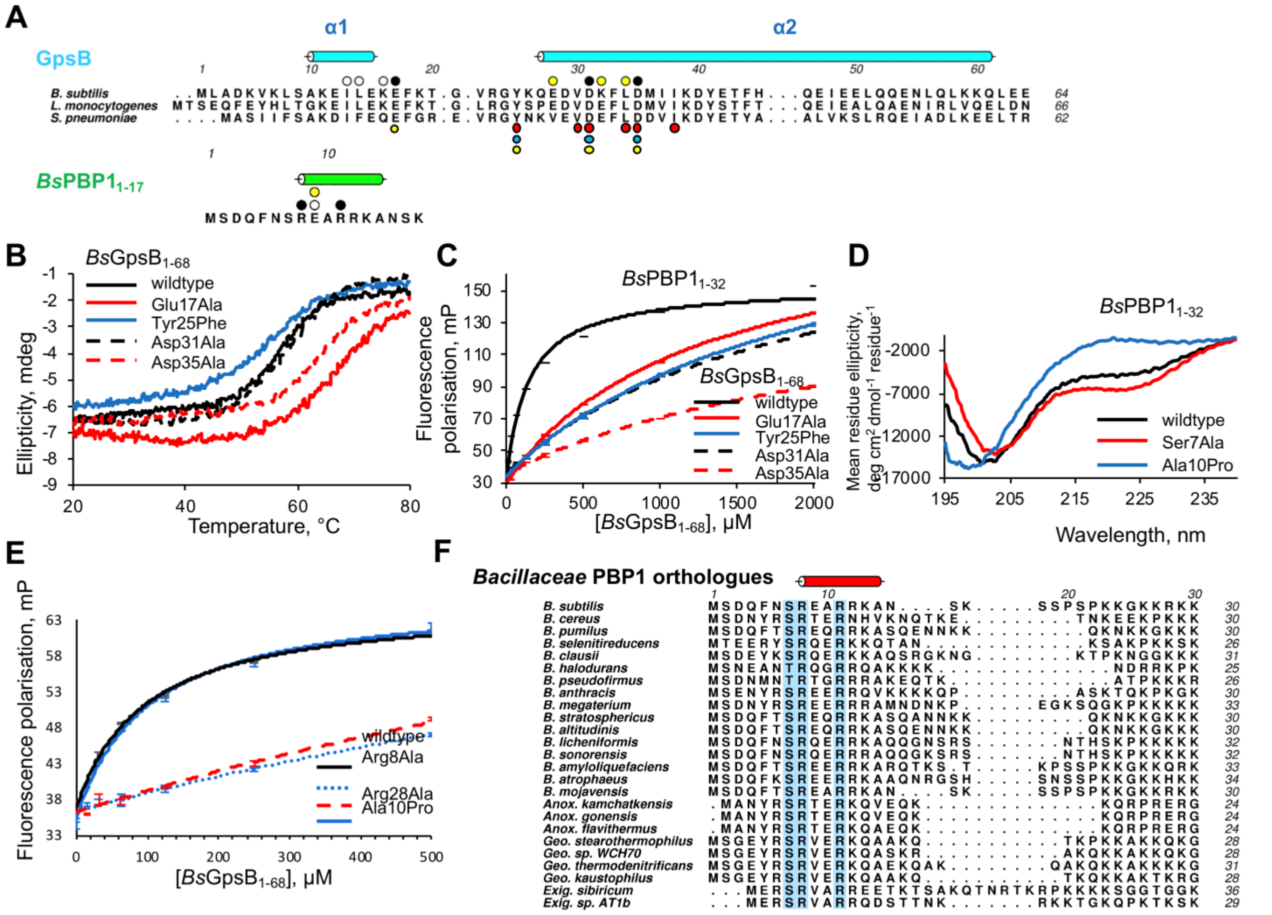

Supplementary Figure 1. Biophysical effects of mutations in *Bs*GpsB and *Bs*PBP1.
(**A**) A sequence alignment of GpsB proteins with the intermolecular interactions from the *Bs*GpsB$_{5\text{-}64}$:*Bs*PBP1$_{1\text{-}17}$ complex highlighted above: black filled circles - residues in hydrogen bonds or salt bridges; yellow circles - residues in van der Waals' interactions; black unfilled circles – interfacial residues utilising main chain atoms; coloured circles below - residues mutated in this study: *Sp*GpsB Y23, V27, L32, D33, I36 (red); *Lm*GpsB Y27, D33 and D37 (cyan); *Bs*GpsB E17, Y25, D31, D33 (yellow). *Bs*GpsB α-helices α1 and α2 are denoted as cyan cylinders. The sequence of *Bs*PBP1$_{1\text{-}17}$ peptide (below) is annotated as above and the helical region denoted as a green cylinder. (**B**) The wild-type folding of *Bs*GpsB$_{1\text{-}68}$ variants was confirmed by circular dichroism. Protein secondary structure unfolding was monitored with the CD ellipticity signal at 222 nm as a function of temperature. (**C**) Alanine substitutions of conserved asparate and glutamate residues in *Bs*GpsB$_{1\text{-}68}$ cannot bind to TAMRA-labelled wild-type *Bs*PBP1$_{1\text{-}32}$ as measured by fluorescence polarisation. The K_d values are tabulated in **Supplementary Table 1**. (**D**) The helical content of *Bs*PBP1$_{1\text{-}32}$ peptides provides a molecular rationale for the reduced affinity of *Bs*PBP1$_{1\text{-}32}$ variants for *Bs*GpsB. The absolute molar ellipticity signal at 222 nm, which is linearly proportional to helix content[17], was measured by circular dichroism for *Bs*PBP1$_{1\text{-}32}$ peptides. (**E**) The position of the fluorophore does not affect the interaction of labelled *Bs*GpsB$_{1\text{-}32}$ peptides with *Bs*GpsB$_{1\text{-}68}$. A peptide labelled at the C-terminus with fluorescein binds *Bs*GpsB$_{1\text{-}68}$ with the same affinity as a peptide labelled with TAMRA at Ser16Cys (**Figure 1D** and **Supplementary Table 1**). (**F**) A sequence alignment of the cytoplasmic minidomains of representative *Bacillaceae* PBP1 orthologues highlights the importance of the invariant Ser7, Arg8 and Arg11 (blue highlight) for GpsB-binding. The helical region of PBP1 is depicted above by a red cylinder. These sequences are representatives from the branches of a *Bacillaceae* family phylogenetic tree[18]. Exceptions lacking a PBP1 orthologue with a SRxxR(R/K) motif include *Brevibacillus brevis*, *Paenibacillus sp.*, *Lysinibacillus sphaericus*, *Oceanobacillus iheynensis* and *Geobacillus sp.* strains C56_T3 and Y412MC61.

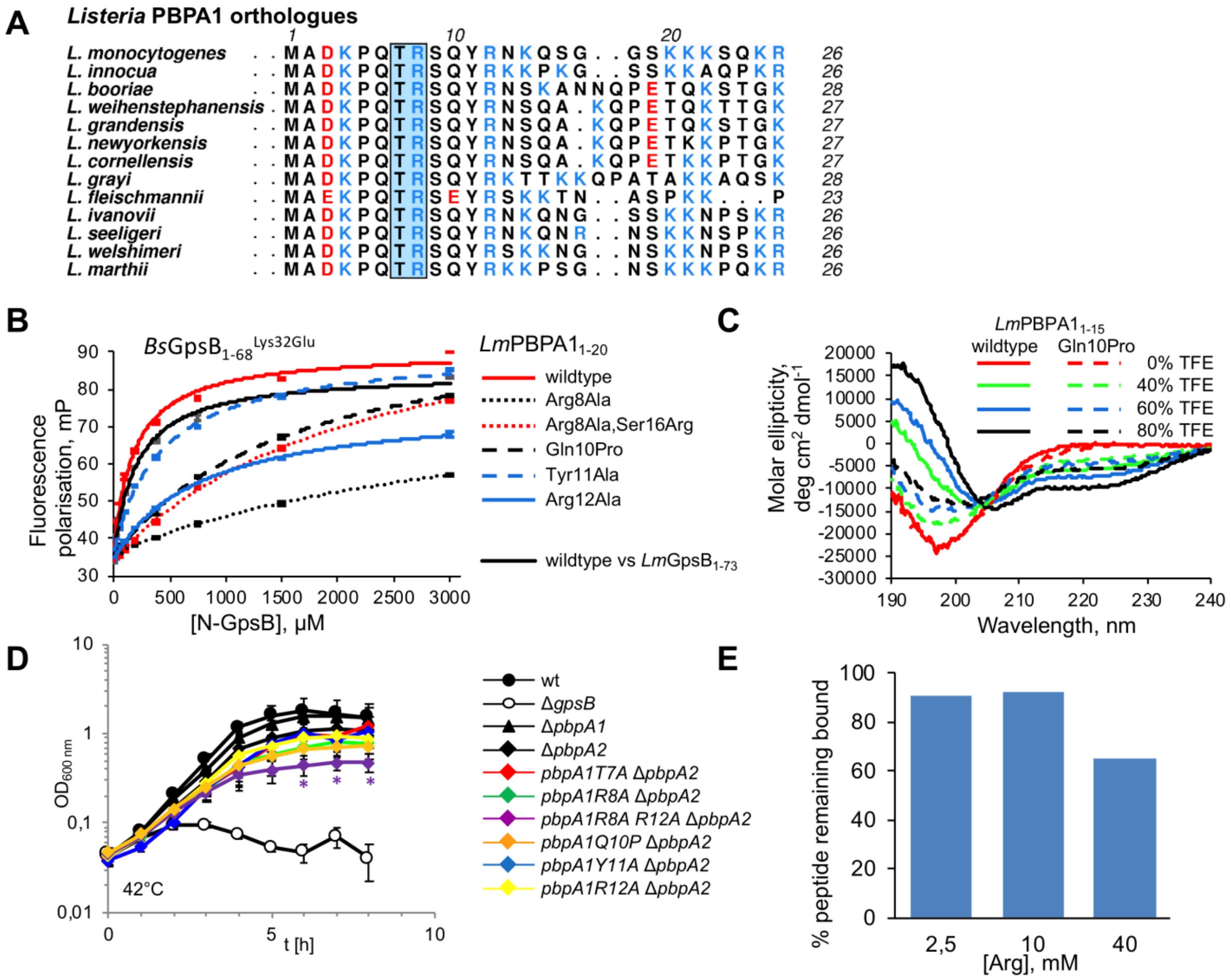

Supplementary Figure 2: *Lm*PBPA1:*Lm*GpsB interactions depend on a conserved arginine. (**A**) The *Lm*PBPA1 TRSQYRN motif is conserved in all publicly available *Listeria* sequences. Basic amino acids (cyan) are more abundant than negatively-charged residues (red). *Lm*PBPA1^{Thr7} and *Lm*PBPA1^{Arg8} are highlighted in blue. (**B**) *Lm*PBPA1$_{1\text{-}20}^{Arg8}$ is the most critical *Lm*GpsB$_{1\text{-}73}$ binding determinant. The binding of fluorescein-labelled *Lm*PBPA1$_{1\text{-}20}$ variants to *Lm*GpsB$_{1\text{-}73}$ and its surrogate, *Bs*GpsB$_{1\text{-}68}^{Lys32Glu}$ was monitored by FP. The cognate *Lm*GpsB$_{1\text{-}73}$:*Lm*PBPA1$_{1\text{-}20}$ interaction is represented by the solid black curve; all other interactions involve the *Bs*GpsB$_{1\text{-}68}^{Lys32Glu}$ surrogate. The calculated dissociation constants are listed in **Supplementary Table 1**. (**C**) In the presence of the helix-stabilizing additive, trifluoroethanol (TFE), wild-type *Lm*PBPA1$_{1\text{-}15}$ has a greater helical character than *Lm*PBPA1$_{1\text{-}15}^{Gln10Pro}$. (**D**) Mutations in the cytoplasmic minidomain of *pbpA1* have little impact on growth of a Δ*pbpA2* mutant at 42°C. *L. monocytogenes* contains two bi-functional PBPs, PBPA1 and PBPA2[19], but at least one is requored for viability[5]. If the *Lm*PBPA1:*Lm*GpsB interaction is essential for PBPA1 function, the Thr7Ala, Arg8Ala, Tyr11Ala and Arg12Ala exchanges in *pbpA1* might not be tolerated with a *pbpA2* deletion. However, strains LMS219 (*pbpA1T7A* Δ*pbpA2*), LMS220 (*pbpA1R8A* Δ*pbpA2*), LMS221 (*pbpA1Y11A* Δ*pbpA2*), LMS222 (*pbpA1R12A* Δ*pbpA2*), LMS232 (*pbpA1R8A R12A* Δ*pbpA2*) and LMS233 (*pbpA1Q10P* Δ*pbpA2*) are viable and even grow in BHI broth at 42°C. The only significant growth defect (marked with asterisks, $P<0.01$, *t*-test) was observed for strain LMS232 (*pbpA1R8A R12A* Δ*pbpA2*) in comparison to the *pbpA2* null mutant. Strains EGD-e (wt), LMJR19 (Δ*gpsB*), LMS57 (Δ*pbpA1*) and LMS64 (Δ*pbpA2*) were included as controls. (**E**) The free amino acid L-arginine does not displace fluorescein-labelled *Lm*PBPA1$_{1\text{-}20}$ peptide from *Lm*GpsB$_{1\text{-}73}$ even when present at more than 100-fold excess relative to the *Lm*GpsB$_{1\text{-}73}$ protein. In this experiment, the fluorescein-labelled peptide is at 40 nM concentration and the *Lm*GpsB$_{1\text{-}73}$ protein at 200 µM.

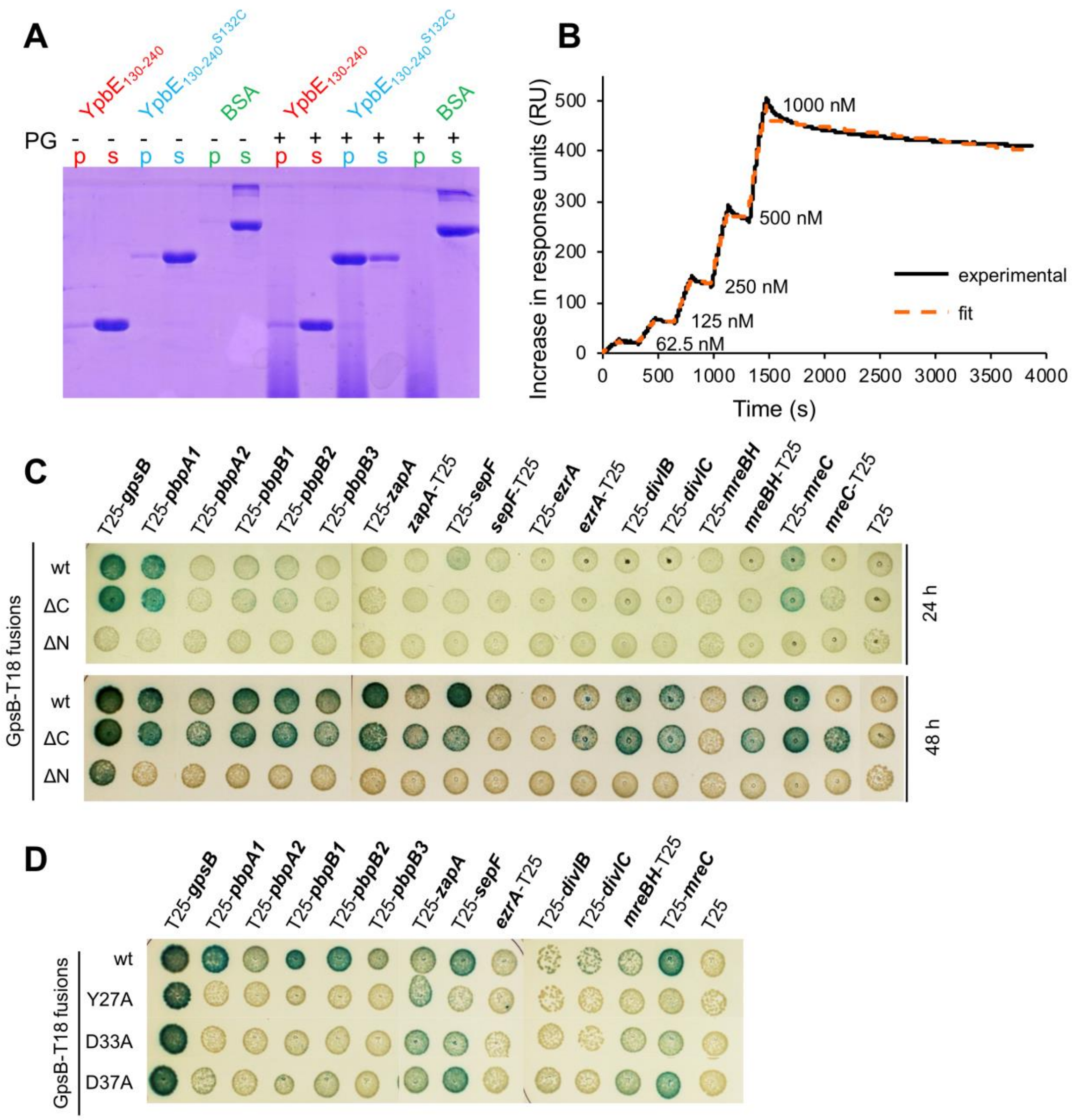

Supplementary Figure 3: Extending the GpsB interactome in *B. subtilis* and *L. monocytogenes*
(**A**) The LysM domain of *Bs*YpbE binds peptidoglycan as a dimer. Almost all the monomeric LysM domain of *Bs*YpbE, *Bs*YpbE$_{130\text{-}240}$; a disulphide cross-linked dimeric form of *Bs*YpbE, *Bs*YpbE$_{130\text{-}240}^{Ser132Cys}$; and the BSA control is found in the supernatant (s) in the absence of PG (left hand side of the SDS-PAGE gel). By contrast, the majority of dimeric *Bs*YpbE$_{130\text{-}240}^{Ser132Cys}$ is found in the pellet (p) in the presence of PG whereas monomeric *Bs*YpbE$_{130\text{-}240}$ and BSA are still found in the supernatant. The blue smear towards the bottom of the gel is PG that has been pelleted by centrifugation. (**B**) *Bs*YrrS interacts directly with *Bs*PBP1. SPR sensorgram of serial injections of increasing concentrations (62.5 nM – 1 µM) of *Bs*YrrS$_{\Delta13\text{-}16}$ over a *Bs*PBP1-immobilised chip surface. The black dashed line represents the reference-subtracted sensorgrams and the red-dashed line represents the fit using single-cycle kinetics with a 1:1 binding model yielding a dissociation constant of 20 ± 0.2 nM. *Bs*YrrS$_{\Delta13\text{-}16}$ was used to reduce non-specific binding to the chip surface. (**C**) The interaction of *Lm*GpsB with selected cell division proteins is dependent upon the *Lm*GpsB N-terminal domain. The agar plates in this BACTH of full-length *Lm*GpsB and variants lacking its N- (ΔN) or C-terminal domain (ΔC) against cell division proteins were photographed after 24 and 48 hrs at 30°C. pKT25 (T25) without a fusion partner was used as a negative control. (**D**) Tyr27, Asp33 and Asp37 in *Lm*GpsB are essential for some, but not all, of the interactions against selected cell division proteins. The agar plates in this BACTH were photographed after 48 hrs at 30°C.

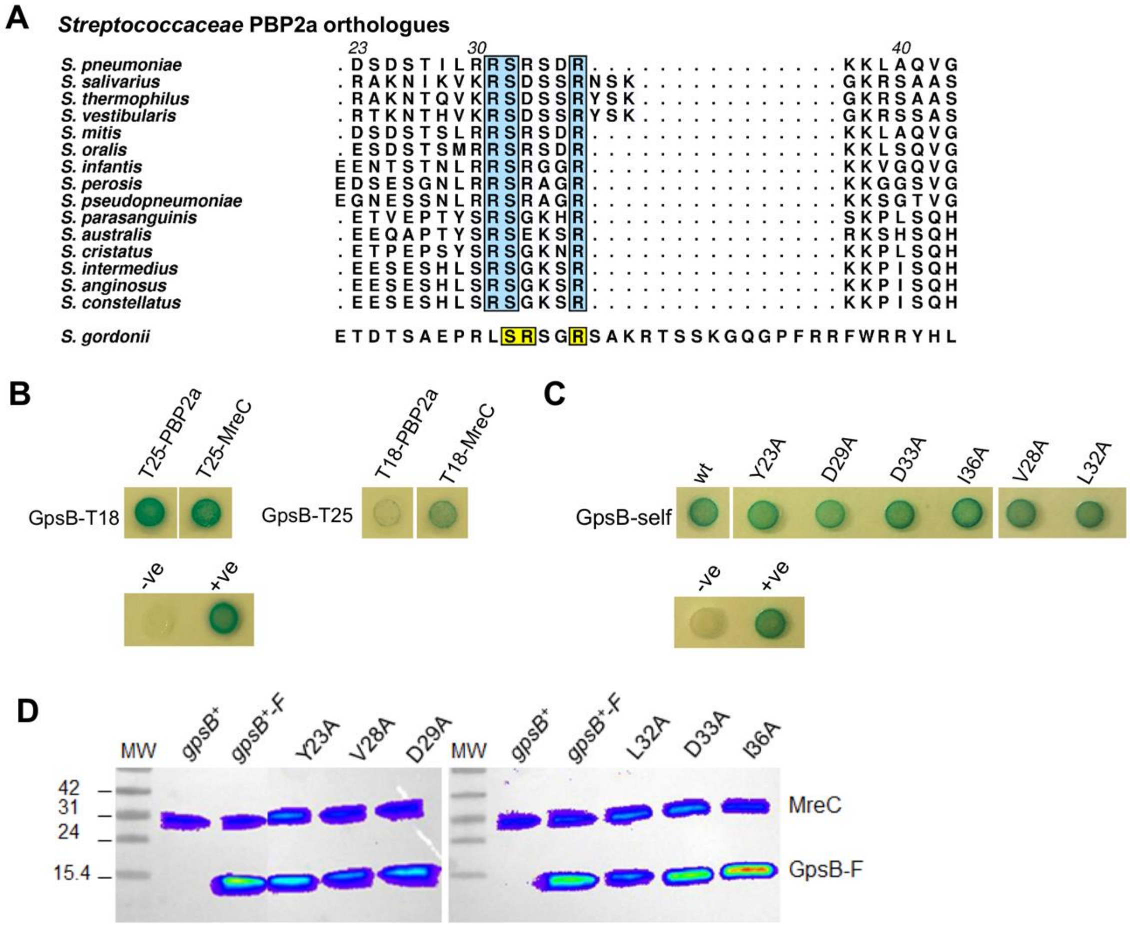

Supplementary Figure 4. *Sp*GpsB mutations affect *Sp*PBP2a-binding but not expression. (**A**) Sequence alignment of *Streptococcaceae* PBP2a orthologues reveals that Arg31, Ser32 and Arg36 (blue highlights) and the RSxxxR motif (boxed) are conserved. The conservation of Ser32 reflects either its interaction with *Sp*GpsB^{Asp33} and/or its stabilization of the type I β-turn between Arg30 and Arg33 in *Sp*GpsB$_{1\text{-}63}$:*Sp*PBP2a$_{23\text{-}45}$ (molecule 1). The serine/glycine distribution is consistent with preferential occupancies of the *i* and *i*+2 (serine) and *i*+3 (glycine) positions in type I β-turns[20]. A diverse set of sequences were selected based on a phylogenetic tree of *Streptococci*[21]. The RSxxxR motif is conserved in the *mitis*, *salivarius* and *anginosus* subgroups but not in *bovis*, *mutans* and *pyogenes*. *S. gordonii*, in the *mitis* subgroup, is an exception and encodes SRSGR (yellow highlights, below) that is a near perfect match to the SRxxR(R/K) motif in *Bacillaceae* PBP1 proteins. (**B**) The reciprocal BACTH analysis of the *Sp*GpsB-T25 data in **Figure 5A** confirms the interactions of *Sp*GpsB with *Sp*PBP2a and *Sp*MreC. The agar plates were photographed after 40 hrs incubation at 30°C. (**C)** BACTH of the self-interactions of *Sp*GpsB variants reveals that all proteins retain the ability to interact implying that the mutations do not affect protein folding. The agar plates were photographed after 40 hrs incubation at 30°C. (**D**) *Sp*GpsB-FLAG variants are expressed at wild-type levels. Western analyses of *S. pneumoniae* strains showing the expression of *Sp*GpsB-FLAG variants, detected with an anti-FLAG antibody as described in the Methods. Anti-MreC was performed as a loading control. Strains used are *gpsB*$^+$ (IU11488, *gpsB*$^+$-P$_c$-*erm*//P$_{Zn}$-*gpsB*$^+$), *gpsB*$^+$-F (IU13442, *gpsB*$^+$-FLAG-P$_c$-*erm*//P$_{Zn}$-*gpsB*$^+$), Y23A (IU13364, *gpsB* Y23A-FLAG-P$_c$-*erm*//P$_{Zn}$-*gpsB*$^+$), V28A (IU13366, *gpsB* V28A-FLAG-P$_c$-*erm*//P$_{Zn}$-*gpsB*$^+$), D29A (IU13368, *gpsB* D29A-FLAG-P$_c$-*erm*//P$_{Zn}$-*gpsB*$^+$), L32A (IU13370, *gpsB* L32A-FLAG-P$_c$-*erm*//P$_{Zn}$-*gpsB*$^+$), D33A (IU13372, *gpsB* D33A-FLAG-P$_c$-*erm*//P$_{Zn}$-*gpsB*$^+$), and I36A (IU13374, *gpsB* I36A-FLAG-P$_c$-*erm*//P$_{Zn}$-*gpsB*$^+$). The expected molecular mass of GpsB-FLAG is 13.7 kDa.

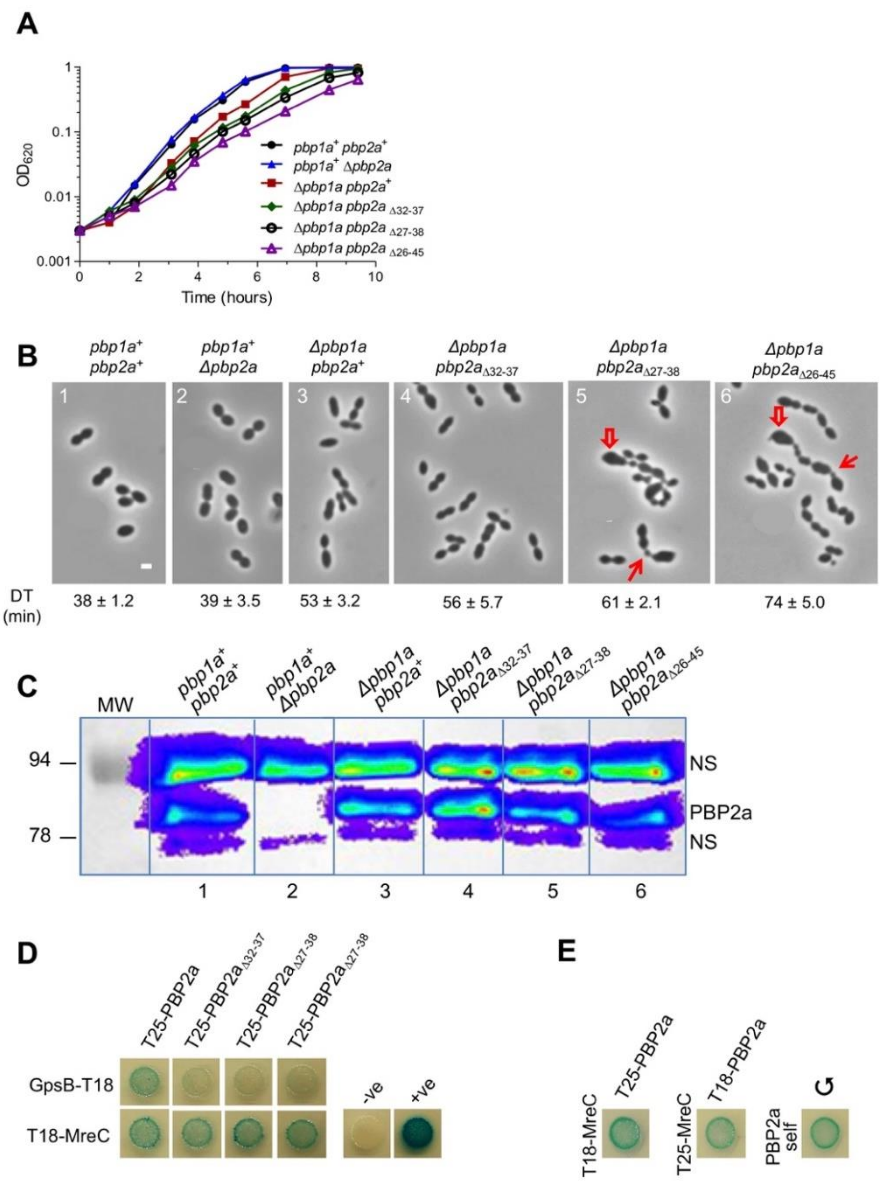

Supplementary Figure 5. Phenotypes of mutant strains lacking the *Sp*PBP2a RSxxxR motif. Truncation of residues 27-38 or 26-45 of *Sp*PBP2a in a Δ*pbp1a* genetic background results in longer doubling times and abnormal morphologies. Representative growth curves (**A**) and phase-contrast micrographs (**B**) of *S. pneumoniae* strains IU1824 (D39 Δ*cps* rpsL1 parent), IU13256 (Δ*pbp*2a), IU13444 (Δ*pbp1*a), IU13446 (*pbp2a*$_{\Delta 32\text{-}37}$ Δ*pbp1a*), IU13448 (*pbp2a*$_{\Delta 27\text{-}38}$ Δ*pbp1a*) and IU13450 (*pbp2a*$_{\Delta 26\text{-}45}$ Δ*pbp1a*); doubling times are reported below (**B**). Deleting *Sp*PBP2a residues 27-38 or 26-45 in a Δ*pbp*1a background resulted in highly variable cell sizes: wide and narrow arrows point to abnormally large and small cells, respectively. All micrographs were taken at mid exponential phase (OD$_{620}$ ≈ 0.15) and are at the same magnification (scale bar = 1 μm). (**C**) The expression of *Sp*PBP2a truncated variants is like wild-type. Western analyses of *S. pneumoniae* strains IU1824 (*pbp1a*$^{+}$, *pbp2a*$^{+}$), IU13256 (*pbp1a*$^{+}$, Δ*pbp2a*), IU13444 (Δ*pbp1a*, *pbp2a*$^{+}$), IU13446 (Δ*pbp1a*, *pbp2a*$_{\Delta 32\text{-}37}$), IU13448 (Δ*pbp1a*, *pbp2a*$_{\Delta 27\text{-}38}$) and IU13450 (Δ*pbp1a*, *pbp2a*$_{\Delta 26\text{-}45}$). The expected molecular masses are 80.9 kDa (WT *Sp*PBP2a), 80.2 kDa (*Sp*PBP2a$_{\Delta 32\text{-}37}$), 79.4 kDa (*Sp*PBP2a$_{\Delta 27\text{-}38}$) and 78.5 kDa (*Sp*PBP2a$_{\Delta 26\text{-}45}$). NS indicate non-specific bands that were also present in the Δ*pbp2a* strain. (**D**) Truncation of the RSxxxR GpsB-binding motif in *Sp*PBP2a results in a progessive decrease in interaction with *Sp*GpsB but not with *Sp*MreC. The interactions of the *Sp*PBp2a Δ32-37, Δ27-38, Δ26-45 truncated variants with *Sp*GpsB and *Sp*MreC were analysed by BACTH. Samples were photographed after 36 hrs incubation at 30°C. (**E**) *Sp*PBp2a interacts directly with *Sp*MreC in BACTH. Samples were photographed after 40 hrs incubation at 30°C.

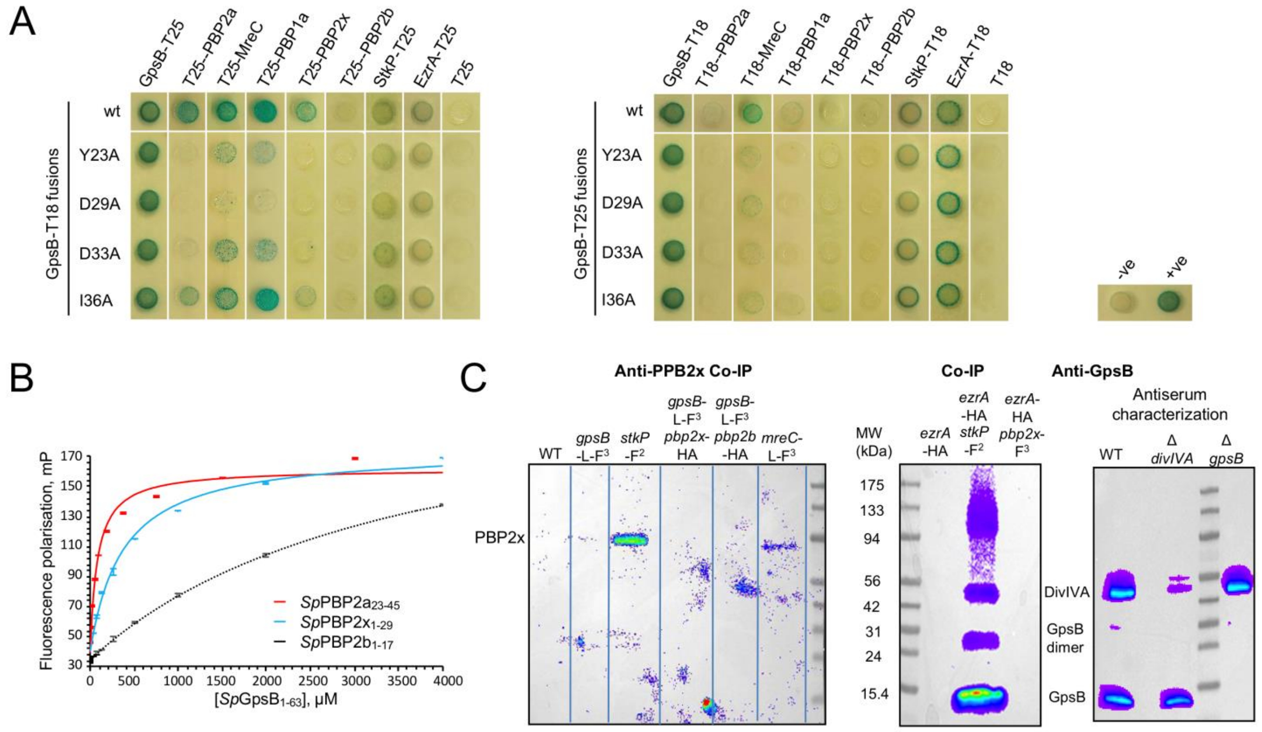

D

Interaction of *Sp*GpsB with other proteins as revealed by different assays

	PBP2a	MreC	PBP1a	PBP2x	PBP2b	StkP	EzrA
BACTH	+++	+++	+++	++	-	+[a]	+[a]
FP	+++	nd	nd	++	-	nd	nd
Co-IP	++[a]	++[a]	+[a]	-[a]	++[a]	++[a]	++[a]

+++: strong signal, ++: signal present, +: weak signal, -: signal absent, nd: not determined.
[a]: reported in Rued *et al.*, 2017

Supplementary Figure 6. *Sp*GpsB interacts with different cell division proteins.
(**A**) *Sp*GpsB interacts with *Sp*PBP1a, *Sp*PBP2x, *Sp*StkP and *Sp*EzrA, as well as *Sp*MreC and *Sp*PBP2a, but not with *Sp*PBP2b, as detected by BACTH. Tyr23, Asp29, Asp33 and Ile36 are essential for some, but not all, of the interactions against these cell division proteins. The agar plates were photographed after 40 hrs at 30°C. Some of the interactions of *Sp*GpsB and its allelic variants in **Figure 5A** are reproduced here for the sake of comparison and consistency with **Supplementary Figure 3D**. (**B**) By FP *Sp*GpsB$_{1-63}$ interacts with TAMRA-labelled *Sp*PBP2a$_{23-45}$ (red) and fluorescein-labelled *Sp*PBP2x$_{1-29}$ (blue), but not fluorescein-labelled *Sp*PBP2b$_{1-17}$ (black). The relevant dissociation constants are listed in **Supplementary Table 1**. (**C**) Left panel, pairwise co-IP detection of *Sp*PBP2x with *Sp*StkP-F^2 and *Sp*MreC-L-F^3, but not with *Sp*GpsB-L-F^3 using anti-PBP2x to detect *Sp*PBP2x (prey) complexed with FLAG-tagged proteins. Strains used were IU1945 (WT), IU5458 (*gpsB*-L-F^3), IU7434 (*stkP*-F^2), IU11314 (*gpsB*-L-F^3 *pbp2x*-HA), IU11316 (*gpsB*-L-F^3 *pbp2b*-HA), and IU4970 (*mreC*-L-F^3). The expected molecular mass of *Sp*PBP2x is 82.4 kDa. Middle panel, pairwise co-IP detection of *Sp*GpsB and *Sp*DivIVA with *Sp*StkP-F^2, but not with *Sp*PBP2x-F^3 using an anti-GpsB serum to detect *Sp*GpsB and *Sp*DivIVA (preys) complexed with FLAG-tagged proteins. Strains used were IU6810 (*ezrA*-HA, non-FLAG-tagged control), IU12077 (*ezrA*-HA *stkP*-F^2), and IU11880 (*ezrA*-HA *pbp2x*-F^3). Right panel, characterisation of anti-*Sp*GpsB serum generated from rabbits immunized with purified *Sp*GpsB$_{1-63}$. Lysates were prepared from WT (IU1945), Δ*divIVA* (IU8496) or Δ*gpsB* (IU6442) strains. The band at ~13 kDa is *Sp*GpsB (expected molecular mass is 12.6 kDa) and that at ~50 kDa is *Sp*DivIVA (expected molecular mass is 30.3 kDa, but typically runs at ~50 kDa on SDS-PAGE). (**D**) Summary of the interaction of *Sp*GpsB with other proteins as revealed by BACTH (data from **A**), FP (**B**) and co-IP (**C** and [10]).

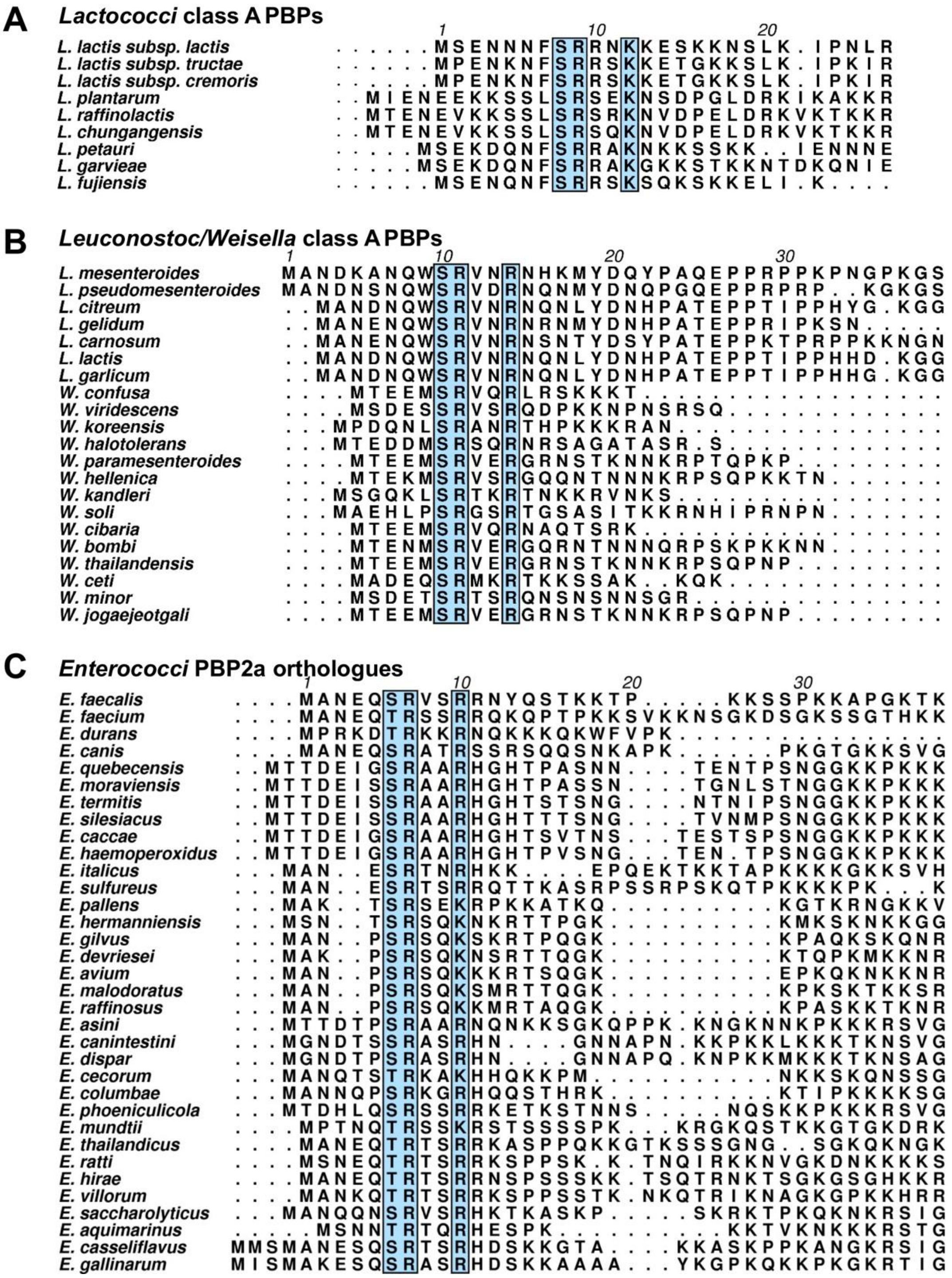

Supplementary Figure 7. The SRxxR(R/K) motif is conserved in other *Lactobacillales* PBPs. Sequence alignments are shown for class A PBPs from *Lactococci* (**A**), *Leuconostoc/Weisella* (**B**) and *Enterococci* (**C**); the *L*. and *W*. prefixes in the species names in panel B correspond to *Leuconostoc* and *Weissella* respectively. The SRxx(R/K) motif is highlighted in blue. Binding and structural studies reported herein are consistent with GpsB binding tolerating either an Arg or a Lys at the underlined position in the SRxxR(R/K) sequence. A phylogenetically diverse set of representative *Leuconstoc/Weisella* and *Enterococci* genomes were chosen based on respective phylogenetic trees[22,23]. The motif is widely conserved in all families, with the exception of *Oenococcus oeni*, *L. fallax* and *L. ficulneum/L. pseudoficulneum/L. fructosum* sub-groups within *Leuconstoc*. This alignment, and all others, was created in ALINE[24].

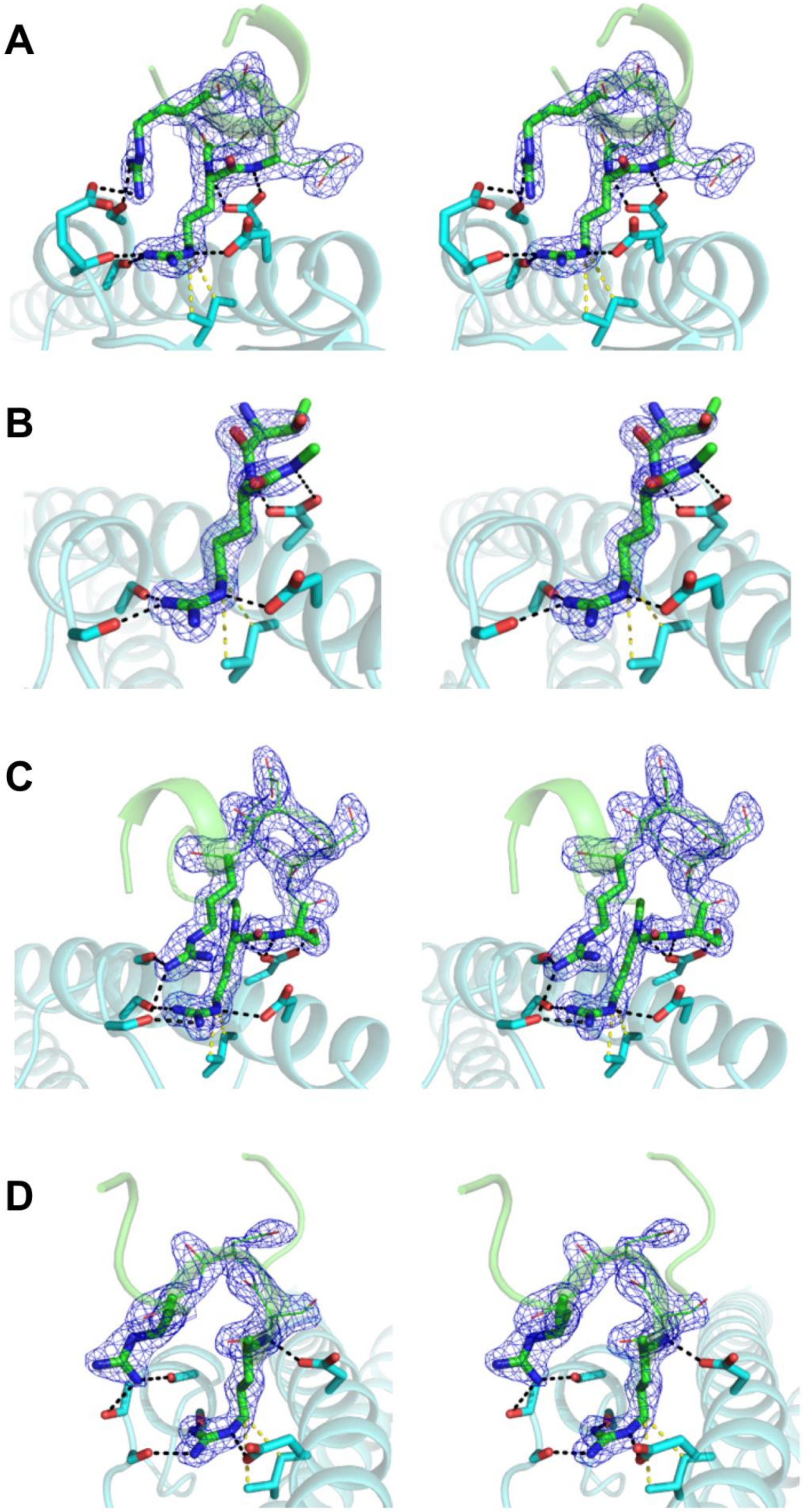

Supplementary Figure 8. Electron density maps for peptides in the GpsB structures. Stereograms of the final, Refmac-weighted 2mF$_{obs}$-DF$_{calc}$ electron density maps for (**A**) *Bs*GpsB$_{5-64}$:*Bs*PBP1$_{1-17}$, contoured at 0.09 electrons per Å^3; (**B**) *Bs*GpsB$_{5-64}^{Lys32Glu}$:*Lm*PBPA1$_{1-15}$, (contoured at 0.14 electrons per Å^3; (**C**) *Sp*GpsB$_{4-63}$:*Sp*PBP2a$_{27-40}$, molecule 1 and (**D**) *Sp*GpsB$_{4-63}$:*Sp*PBP2a$_{27-40}$, molecule 2 (both contoured at 0.5 electrons per Å^3. The colours, interactions and view is the same as in **Figure 2C**; interfacial residues are shown as sticks, other amino acids as lines.

Supplementary References

1. Kumar, S. & Bansal, M. Dissecting alpha-helices: position-specific analysis of α-helices in globular proteins. *Proteins* **31**, 460-476 (1998).
2. Doig, A.J. & Baldwin, R.L. N- and C-capping preferences for all 20 amino acids in α-helical peptides. *Prot. Sci.* **4**, 1325-1336 (1995).
3. Johnson, C.L. et al The antibacterial toxin colicin N binds to the inner core of lipopolysaccharide and close to its translocator protein. *Mol Microbiol.* **92**, 440-452 (2014).
4. Rismondo, J. et al. Structure of the bacterial cell division determinant GpsB and its interaction with penicillin binding proteins. *Mol. Microbiol.* **99**, 978-998 (2016).
5. Rismondo, J., Möller, L., Aldridge, C., Gray, J., Vollmer, W. & Halbedel, S. Discrete and overlapping functions of peptidoglycan synthases in growth, cell division and virulence of *Listeria monocytogenes*. *Mol. Microbiol.* **95**, 332-351 (2015).
6. Lanie, J.A. et al. Genome sequence of Avery's virulent serotype 2 strain D39 of *Streptococcus pneumoniae* and comparison with that of unencapsulated laboratory strain R6. *J. Bacteriol.* **189**, 38-51 (2007).
7. Tsui, H.C. et al. Suppression of a deletion mutation in the gene encoding essential PBP2b reveals a new lytic transglycosylase involved in peripheral peptidoglycan synthesis in *Streptococcus pneumoniae* D39. *Mol. Microbiol.* **100**, 1039-1065 (2016).
8. Land, A.D. et al. Requirement of essential Pbp2x and GpsB for septal ring closure in *Streptococcus pneumoniae* D39. *Mol. Microbiol.* **90**, 939-955 (2013).
9. Land, A.D. & Winkler, M.E. The requirement for pneumococcal MreC and MreD is relieved by inactivation of the gene encoding PBP1a. *J. Bacteriol.* **193**, 4166-4179 (2011).
10. Rued, B.E. et al. Suppression and synthetic-lethal genetic relationships of Δ*gpsB* mutations indicate that GpsB mediates protein phosphorylation and penicillin-binding protein interactions in *Streptococcus pneumoniae* D39. *Mol. Microbiol.* **103**, 931-957 (2017).

11. Tsui, H.C. et al. Pbp2x localizes separately from Pbp2b and other peptidoglycan synthesis proteins during later stages of cell division of *Streptococcus pneumoniae* D39. *Mol. Microbiol.* **94**, 21-40 (2014).

12. Zheng, J.J., Perez, A.J., Tsui, H.T., Massidda, O. & Winkler, M.E. Absence of the KhpA and KhpB (JAG/EloR) RNA-binding proteins suppresses the requirement for PBP2b by overproduction of FtsA in *Streptococcus pneumoniae* D39. *Mol. Microbiol*. **106**, 793-814 (2017).

13. Arnaud, M., Chastanet, A. & Debarbouille, M. New vector for efficient allelic replacement in naturally nontransformable, low-GC-content, gram-positive bacteria. *Appl. Environ. Microbiol.* **70**, 6887-6891 (2004).

14. Karimova, G., Pidoux, J., Ullmann, A. & Ladant, D. A bacterial two-hybrid system based on a reconstituted signal transduction pathway. *Proc. Natl. Acad. Sci. USA* **95**, 5752-5756 (1998).

15. Claessen, D., Emmins, R., Hamoen, L.W., Daniel, R.A., Errington, J. & Edwards, D.H. Control of the cell elongation-division cycle by shuttling of PBP1 protein in *Bacillus subtilis*. *Mol. Microbiol.* **68**, 1029-1046 (2008).

16. Rismondo, J., Wamp, S., Aldridge, C., Vollmer, W. & Halbedel, S. Stimulation of PgdA-dependent peptidoglycan N-deacetylation by GpsB-PBP A1 in *Listeria monocytogenes*. *Mol. Microbiol*. **107**, 472-487 (2018).

17. Chen, Y.H., Yang, J.T. & Martinez, H.M. Determination of the secondary structures of proteins by circular dichroism and optical rotatory dispersion. *Biochemistry* **22**, 4120-4131 (1972).

18. Schmidt, T.R., Scott, E.J. 2nd & Dyer, D.W. Whole-genome phylogenies of the family *Bacillaceae* and expansion of the sigma factor gene family in the *Bacillus cereus* species-group. *BMC Genomics* **12**, 430 (2011).

19. Korsak, D., Markiewicz, Z., Gutkind, G.O. & Ayala, J.A. Identification of the full set of *Listeria monocytogenes* penicillin-binding proteins and characterization of PBPD2 (Lmo2812). *BMC Microbiol.* **10**, 239 (2010).
20. Hutchinson, E.G. & Thornton, J.M. A revised set of potentials for β-turn formation in proteins. *Prot. Sci.* **3**, 2207-16 (1994).
21. Gao, X.Y., Zhi, X.Y., Li, H.W., Klenk, H.P. & Li, W.J. Comparative genomics of the bacterial genus *Streptococcus* illuminates evolutionary implications of species groups. *PLoS One* **9**, e101229 (2014).
22. Chelo, I.M., Zé-Zé, L. & Tenreiro, R. Congruence of evolutionary relationships inside the *Leuconostoc-Oenococcus-Weissella* clade assessed by phylogenetic analysis of the 16S rRNA gene, *dnaA*, *gyrB*, *rpoC* and *dnaK*. *Int. J. Syst. Evol. Microbiol*. **57** 276-286 (2007).
23. Zhong, Z. *et al*. Comparative genomic analysis of the genus *Enterococcus*. *Microbiol Res*. **196**, 95-105 (2017).
24. Bond, C.S. & Schüttelkopf, A.W. ALINE: a WYSIWYG protein-sequence alignment editor for publication-quality alignments. *Acta Crystallogr*. **D65** 510-512 (2009).
